# Supplementary material for: Fine-scale diversity of microbial communities due to satellite niches in boom and bust environments
Source: PLoS Comput Biol. 2022 Dec 27;18(12):e1010244. doi: 10.1371/journal.pcbi.1010244 (PMC9829172; doi:10.1371/journal.pcbi.1010244)
Supplement: S1 Text — Fig A. A detailed view of multi-strain coexistence, similar to Fig 2B. Heatmaps showing the final community diversity (number of strains) as a function of the growth parameters gC and KC. Given a strain C with variable gmax and K along with fixed strains A and B, the heatmap shows the number of coexisting species in the steady state of serial dilutions. Here, the region above the blue line represents where C drives A out of the steady state, and the region above the red line represents where B is driven out by C. The green regions represent areas where more than one strain can coexist. (b) shows a zoomed-in view of the boxed region in (a), highlighting the region where two (light green) and three (dark green) strains can coexist. Fig B. Histograms showing statistics of the number of coexisting strains: (a) when we allow small variations in how a species partitions two resources, with no variation in Monod growth parameters, and (b) when we allow changes in both the Monod parameters as well as resource partitioning. Bar plots on the margins in blue and red show the number of surviving strains from species I and II respectively. In (a), the variation in resource partitioning was as follows: each species’ resource partitioning was independently sampled from a normal distribution, N(0.2,0.12) for species I and N(0.7,0.12) for species II. Fig C. Growth curve of the simplified model. The 3 solid lines show how growth rate depends on the resource concentration in our simplified model. Here gA, gB and gC are approximations of the maximum growth rate from Monod’s law of growth, and KA, KB and KC are approximations of the substrate affinity. Fig D. Monod growth curve approximated by the simplified model. The grey line shows the growth curve of a strain that obeys Monod’s law of growth on a single resource, with maximum growth rate gAMonod and affinity KAMonod. The blue line is the growth growth curve of a strain that grows on a set of unique resources and follows our simplifi [file pcbi.1010244.s001.pdf]

S1 Text

1 Monod's growth allows for the satellite niche formation

1.1 Serial dilution vs constant nutrient supply: why, in the former case, the competitive exclusion principle can be violated

The competitive exclusion principle is usually thought to restrict the number of species surviving in the environment to be less or equal to that of the unique resources. (Barring crossfeeding and other types of interaction which can be understood as providing additional, positive or negative, resource.) However, it is also known that the species growing according to the Monod's law can be either "r-strategists" maximising their growth rate on a given source when it is supplied in plentiful amounts or "K-strategists" striving to survive on lesser amounts of resource whenever the supply is scarce. While in the case of constant nutrient supply (chemostat) the number of species that grow under the Monod's dynamics can never exceed the number of (generalized, i. e. including the bacteriophages as negative resources, effects of crossfeeding etc) resources, the environments that resemble the serial dilution experimental setting under certain supply regimes may allow for the survival of more than one species on a single resource.

This can happen because the Monod's dynamics, under which the growth rate for a species B_i is given by $g_i\, c/(c + K_i)$ (with g_i, K_i, c being the Monod's parameters of the microbe's and the nutrient's current abundance, respectively) may favor, say, first B_1, when the nutrient is still comparatively abundant, and then B_2 when the nutrient is closer to being depleted. When we are talking serial dilution, it is important for a species' successful invasion to make sure that its abundance gets multiplied at least $D-$fold during a single dilution cycle (D being the dilution coefficient). Rephrasing this requirement in terms of classic ecology, we would say that the invasion fitness [1] of an invading species is in our case its multiplification coefficient achieved at the end of the first dilution cycle divided by D, so that when it is less than 1 the species cannot invade the environment in a seed amount, when the effect of its presence on the system dynamics is negligible, while when it is greater than 1 the species invades successfully. Comparing the two multiplication rates we see that, if $g_1 > g_2$,

$$\frac{g_1\, c}{c + K_1} > \frac{g_2\, c}{c + K_2} \;\Leftrightarrow\; c > c^* = \frac{g_2\, K_1 - g_1\, K_2}{g_1 - g_2}, \tag{S1}$$

so that at $K_1/K_2 > g_1/g_2$ there is a turning point $c = c^*$ from (S1). When, during the dilution cycle, the nutrient concentrations drops below that point, B_2 starts multiplying faster than B_1 and thus, in total, under some conditions may yet succeed in growing $D-$fold simultaneously with B_1. No degeneration (specific functional dependence) in terms of g, $K-$parameters is required for the coexistence to be achieved: even if the region of coexistence is narrow, it is still not a curve in the g, K-plane defined by some specific function $g = g(K)$, but rather a region of parameters.

1.2 Coexistence of two species on a single resource: the criterion

Now we proceed at deriving the formal criterion of the necessary and sufficient conditions under which two microbes obeying the Monod's growth law can coexist on a single source in a serial dilution setting.

Consider a serial dilution setting. Let the resource c be supplied in the amount c_0 in the beginning of each dilution cycle, long enough for it to get effectively depleted in the end by the microbe or microbes invading the environment. Let the bacteria B_1 with the maximum growth rate g_1 and the $K-$parameter K_1 establish a monoculture steady state in this environment. During each dilution cycle, the microbe will grow, so that

$$\frac{dN_1}{dt} = \frac{g_1\, c}{c + K_1}\, N_1. \tag{S2}$$

The resource c gets depleted as the microbe grows. For the sake of simplicity we set the yield equal to 1, so that

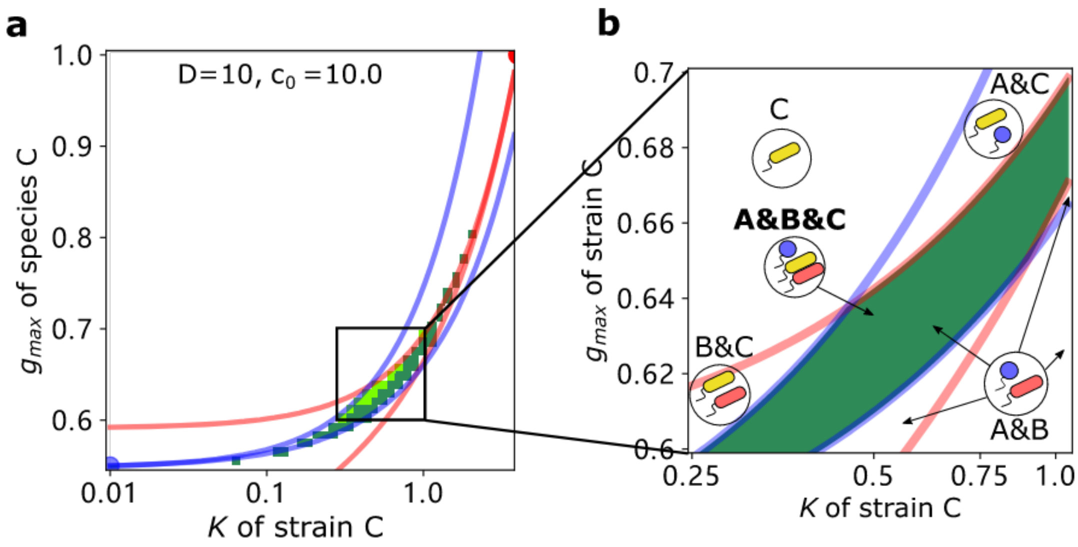

Figure A: A detailed view of multi-strain coexistence, similar to Fig 2B. Heatmaps showing the final community diversity (number of strains) as a function of the growth parameters g_C and K_C. Given a strain C with variable g_{max} and K along with fixed strains A and B, the heatmap shows the number of coexisting species in the steady state of serial dilutions. Here, the region above the blue line represents where C drives A out of the steady state, and the region above the red line represents where B is driven out by C. The green regions represent areas where more than one strain can coexist. (b) shows a zoomed-in view of the boxed region in (a), highlighting the region where two (light green) and three (dark green) strains can coexist.

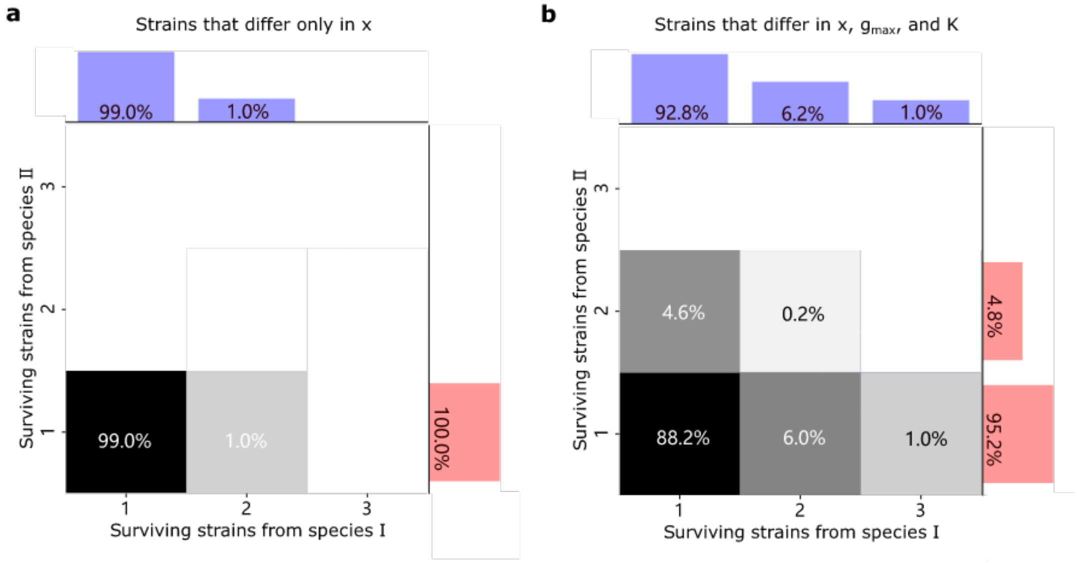

Figure B: Histograms showing statistics of the number of coexisting strains: (a) when we allow small variations in how a species partitions two resources, with no variation in Monod growth parameters, and (b) when we allow changes in both the Monod parameters as well as resource partitioning. Bar plots on the margins in blue and red show the number of surviving strains from species I and II respectively. In (a), the variation in resource partitioning was as follows: each species' resource partitioning was independently sampled from a normal distribution, $\mathcal{N}(0.2, 0.1^2)$ for species I and $\mathcal{N}(0.7, 0.1^2)$ for species II.

$$\frac{dc}{dt} = -\frac{dN_1}{dt} = -\frac{g_1\, c}{c+K_1}\, N_1 = -\frac{g_1\, c}{c+K_1}\, (c^{(tot)} - c), \tag{S3}$$

where the constant $c^{(tot)}$, representing total "carbon units" present in the medium, comes from the conservation law (valid, separately, for each dilution cycle):

$$c + N_1 = c_0 + N_{1,\,0} = c^{(tot)} = \text{const}, \tag{S4}$$

$N_{1,\,0}$ being the initial abundance of the microbe at the beginning of a dilution cycle.

If the dilution cycle is long enough, then for the steady monoculture state we can write effectively

$$\int_0^\infty \frac{d\,\log(N_1/N_{1,\,0})}{dt}\, dt = \int_0^\infty \frac{g_1\, c}{c+K_1}\, dt = \log D, \tag{S5}$$

as the multiplication coefficient (the number by which the initial abundance is multiplied towards the end of each dilution cycle) should be equal to the dilution coefficient D. For the same reason the steady state initial abundance of the microbe for each dilution cycle should be

$$N_{1,\,0} = \frac{c_0}{D-1}, \tag{S6}$$

as can be seen using (S4). Indeed, in the beginning of a cycle in the steady state $c^{(tot)} = c_0 + N_{1,\,0}$ and in the end it is $c^{(tot)} = DN_{1,\,0}$, hence $c_0 + N_{1,\,0} = DN_{1,\,0}$ which is tantamount to (S6). Thus the constant $c^{(tot)}$ appearing in (S4) in the steady state is found to be equal

$$c^{(tot)} = \frac{D\, c_0}{D-1} \tag{S7}$$

If a microbe B_2 is able to invade the monoculture environment (shaped by B_1) in the seed amount, then through its first dilution cycle in the environment it should grow more than $D-$fold.

$$\int_0^\infty \frac{g_2\, c}{c+K_2}\, dt > \log D, \tag{S8}$$

As, upon introduction into the environment, the invader can influence the dependence $c(t)$ in but a negligibly small way, we can use (S3) to give dc/dt as we rewrite (S8) changing variables and going from integration over t to integration over c:

$$\int_0^\infty \frac{g_2\, c}{c+K_2}\, dt = \int_{c(0)}^{c(\infty)} \frac{g_2\, c}{c+K_2}\, \frac{dt}{dc}\, dc = \frac{g_2}{g_1} \int_0^{c_0} \frac{c+K_1}{c+K_2}\, \frac{1}{c^{(tot)} - c}\, dc > \log D, \tag{S9}$$

which, upon integration, together with (S7), yields the condition under which the species B_2 can invade the monoculture environment shaped by B_1:

$$\frac{g_2}{g_1} \Big(\frac{c^{(tot)} + K_1}{c^{(tot)} + K_2}\, \log D + \frac{K_1 - K_2}{c^{(tot)} + K_2}\, \log\, \frac{c_0 + K_2}{K_2} \Big) > \log D. \tag{S10}$$

The condition under which B_1, in its turn, can invade the monoculture environment shaped by the microbe B_2 is obtained from (S10) by swapping the indices 1 and 2. Thus, for a given B_1 with the Monod parameters (g_1, K_1) for each $K_2 < K_1$ there is a range from which g_2 can be chosen so that B_1 and B_2 can share the environment. (Indeed, if both B_2 can invade the monoculture of B_1 and B_1 can invade the monoculture of B_2, no one of the microbes will be able to outcompete the other, so that they would have to share and coexist.) The said range is given by the following:

$$\frac{(c^{(tot)}+K_2)\,\log D}{(c^{(tot)}+K_1)\,\log D+(K_1-K_2)\log\dfrac{c_0+K_2}{K_2}} < \frac{g_2}{g_1} < \\ < \frac{(c^{(tot)}+K_2)\,\log D-(K_1-K_2)\log\dfrac{c_0+K_1}{K_1}}{(c^{(tot)}+K_1)\,\log D}. \tag{S11}$$

Note that, as $g_2 < g_1$, the width of the range of the value $0 < g_2/g_1 < 1$ favouring coexistence should be evaluated with respect to 1.

1.3 Do yields matter?

When bacterial strains or species B_i have different yields, the dynamics of a dilution cycle, say, for B_1-monoculture should be represented as

$$\frac{d\,N_1}{d\,t} = \frac{g_1\,c}{c+K_1}\,N_1, \\ \frac{d\,c}{d\,t} = -\frac{g_1\,c}{c+K_1}\,\frac{N_1}{Y_1}. \tag{S12}$$

Thus the yield-sensitive conservation law goes as

$$c+\frac{N_1}{Y_1} = c^{(tot)} = \text{const}, \tag{S13}$$

which holds, again, for any dilution cycle. In particular,

$$c_0+\frac{N_{1,\,0}}{Y_1} = 0+\frac{D\,N_{1,\,0}}{Y_1}, \tag{S14}$$

as long as the system has reached its steady state. From (S14) we get

$$N_{1,\,0} = \frac{Y_1\,c_0}{D-1}, \tag{S15}$$

so that

$$c^{(tot)} = \frac{D\,c_0}{D-1}, \tag{S16}$$

the same as in (S7). Thus for dt/dc to be used in calculations determining whether another strain or species $B_2\,(g_2, K_2)$ can invade, from (S12), (S13) and (S16) we get

$$\frac{dt}{dc} = -\frac{c+K_1}{g_1\,c\,(c^{(tot)}-c)}, \tag{S17}$$

with $c^{(tot)}$ the same as in (S7). Thus yield Y_1 disappears from the criterion deciding whether B_2 can invade, $\int (g_2\,c/(c+K_2))\,dt > \log D$, while the yield Y_2 never entered this criterion. Same thing happens when determining whether B_1 can invade B_2-shaped environment: both yields either disappear or never enter the key inequality. Thus, in fact, nonunity yields change nothing in the coexistence criterion for two strains or species. This however may not be the case when dealing with coexistence of three or more strains.

1.4 The "mutation-friendly" limit: two strains of the same species (close to each other in terms of their growth rates) coexistence criterion

In the limit $c_0 \gg K_1 > K_2$, and thus $K_{1,2} \ll c^{(tot)} = \frac{D}{D-1}\,c_0$, the microbes sharing the same niche may do so only if they are very close in terms of their growth rates, and those are neatly tuned to each other. If there is a biological reason for a species to be able to lessen considerably its $K-$

value by some small $\Delta K \ll K$ at the expense of decreasing its own growth rate by a tiny little bit (proportional to $(\Delta K/K)^2$) one can observe two strains of the same species surviving on a single source in an environment with boom and bust cycles. Indeed, in this limit the expansion to the first order in terms of the small parameter $K/c^{(tot)}$ (assuming at first $K_1^2/(c^{(tot)})^2 \ll K_2/c^{(tot)} \lesssim K_1/c^{(tot)}$) applied to the criterion (S11) gives

$$1 - \frac{K_1 - K_2}{c^{(tot)}} \Big(\frac{\log(c_0/K_2)}{\log D} + 1 \Big) < \frac{g_2}{g_1} < 1 - \frac{K_1 - K_2}{c^{(tot)}} \Big(\frac{\log(c_0/K_1)}{\log D} + 1 \Big) \tag{S18}$$

To see this, consider the left boundary l_0 of the coexistence criterion (S11), which has the form $l_0 < g_2/g_1 < r_0$. Again, we take the first order approximation in terms of $K_{1,2}/c^{(tot)} \sim K_{1,2}/c_0$.

$$\begin{aligned}
&\frac{(c^{(tot)} + K_2)\, \log D}{(c^{(tot)} + K_1)\, \log D + (K_1 - K_2)\, \log \dfrac{c_0 + K_2}{K_2}} = \\
&= \frac{c^{(tot)} \Big(1 + \dfrac{K_2}{c^{(tot)}}\Big)\, \log D}{c^{(tot)}\, \log D \Big(1 + \dfrac{K_1}{c^{(tot)}} + (K_1 - K_2)\, \log \dfrac{c_0\,(1 + K_2/c_0)}{K_2}\Big)} \simeq \\
&\simeq \frac{1 + \dfrac{K_2}{c^{(tot)}}}{1 + \dfrac{K_1}{c^{(tot)}} + \dfrac{K_1 - K_2}{c^{(tot)}\, \log D}\, \log \dfrac{c_0}{K_2}} \simeq \\
&\simeq \Big(1 + \frac{K_2}{c^{(tot)}}\Big)\Big(1 - \frac{K_1}{c^{(tot)}} - \frac{K_1 - K_2}{c^{(tot)}\, \log D}\, \log \frac{c_0}{K_2}\Big) \simeq \\
&\simeq 1 - \frac{K_1 - K_2}{c^{(tot)}} \Big(\frac{\log(c_0/K_2)}{\log D} + 1 \Big).
\end{aligned} \tag{S19}$$

In the same way we get the first order approximation in terms of the small parameter for the right boundary r_0 of the coexistence region:

$$r_0 = \frac{(c^{(tot)} + K_2)\, \log D - (K_1 - K_2)\, \log \dfrac{c_0 + K_1}{K_1}}{(c^{(tot)} + K_1)}\, \log D \;\simeq\; 1 - \frac{K_1 - K_2}{c^{(tot)}} \Big(\frac{\log(c_0/K_1)}{\log D} + 1 \Big), \tag{S20}$$

so that the size of the coexistence area becomes

$$r_0 - l_0 \simeq \frac{K_1 - K_2}{c^{(tot)}\, \log D}\, \log \frac{K_1}{K_2}. \tag{S21}$$

The larger K_1/K_2, the wider the coexistence region. It is natural to ask, on the other hand, in which way the width of the coexistence region depends on $\Delta K = K_1 - K_2$ when $\Delta K \ll K_{1,2}$. To be precise, it must be noted that in the above we have implicitly assumed $K_1 - K_2$ to be of the same order as K_1 and K_2; if however $(K_1 - K_2)/c^{(tot)} \sim K_{1,2}^2/c_{(tot)}^2$, then, say, in the calculations in (S19) we should keep all the terms of the second order of smallness. If we do that, however, we get the same result for the size of the coexistence region as in (S21), or rather

$$r_0 - l_0 \simeq \frac{(\Delta K)^2}{K\, c^{(tot)}\, \log D}, \tag{S22}$$

with $K \simeq K_2 \simeq K_1$.

For a given microbe B^* with the Monod parameters (g^*, K^*) the species that can coexist with it for any given initial resource concentration c_0 form a banana-shaped shadow in the $g, K-$plane (see Fig 1D). So in an environment resembling a serial dilution setting rather than a chemostat, whenever the nutrient supply is somewhere in between "feast" and "famine", each niche occupied by a specialist would be accompanied by a shadow of satellite niches, that can be occupied by yet another microbe, or, in principle, even by several other species, as we have shown in this paper.

1.5 Why the coexistence range always exists

It remains to prove that the coexistence range (S11) exists for any reasonable initial abundance c_0 of the resource (we assume $c_0 > K_2$) and for any value of the dilution coefficient $D > 1$. More precisely, one can choose any value of the ratio for a pair of $K-$parameters of the Monod's dynamics, $K_1 > K_2$, $x = K_1/K_2 > 1$, and any value of the initial abundance $c_0 = y\,K_1 = x\,y\,K_2$ in the same $K-$units, and be certain to find the ratio g_2/g_1 of the maximum growth rates of the two species such that, with a given dilution coefficient $D > 1$, they would be able to coexist on that single resource c in the serially diluted environment. This ratio g_2/g_1 would be bounded from above and from below, and any value within the range given by (S11) will work.

If we prove that, we can be justified in expecting the satellite niches to eventually emerge under the favourable conditions in nature. In the laboratory, however, more practical question would be whether, for given bacteria B_1 and B_2, with the Monod parameters (g_1, K_1) and (g_2, K_2) respectively, the appropriate conditions (i. e. the initial resource concentration c_0 and the dilution coefficient D) can be found, so that they may share the environment. The answer seems to be positive if $g_1/g_2 < K_1/K_2$, as we will show later. Now we proceed to proving that a satellite niche can always emerge.

It is worth noting that, with $\gamma = g_2/g_1$,

$$\begin{aligned} &\gamma < 1, \\ &x\gamma > 1, \\ &y > \frac{\gamma\, x - 1}{x\,(1-\gamma)}. \end{aligned} \tag{S23}$$

where the first inequality comes from our assumption that $g_2 < g_1$, while the second and the third follow from Eq.(S1) and the fact that c_0 should be greater than the critical value c^* mentioned in (S1) so that the competing microbes could take turns in growing faster than their counterpart.

The said range will exist if, and only if,

$$\frac{(c^{(tot)} + K_2)\,\log D}{(c^{(tot)} + K_1)\,\log D + (K_1 - K_2)\log\dfrac{c_0 + K_2}{K_2}} < \frac{(c^{(tot)} + K_2)\,\log D - (K_1 - K_2)\log\dfrac{c_0 + K_1}{K_1}}{(c^{(tot)} + K_1)\,\log D}, \tag{S24}$$

meaning simply that the lefthand side of the criterion inequality (S11) is indeed less than its righthand side.

Using x, y introduced above (so that the biomass conservation constant $c^{(tot)} = D\,c_0/(D-1) = (D/(D-1))\,x\,y\,K_2$), we transform the inequality (S24) into

$$\begin{aligned} &\frac{(Dxy + D - 1)\,\log D}{(Dy + D - 1)\,x\,\log D + (D-1)\,(x-1)\,\log(xy+1)} < \\ &< \frac{(Dxy + D - 1)\,\log D - (D-1)\,(x-1)\,\log(y+1)}{(Dy + D - 1)\,x\,\log D} \end{aligned} \tag{S25}$$

This can be transformed by algebraic means into an inequality:

$$f(x,y,D) = \frac{x\,(D\,y + D - 1)\,\log(y+1)}{(D\,x\,y + D - 1)\,\log(x\,y+1)} + \frac{(D-1)\,(x-1)\,\log(y+1)}{(D\,x\,y + D - 1)\,\log D} < 1 \tag{S26}$$

Within the relevant region of the parameters, it is possible to determine the maximum value of $f(x,y,D)$ using elementary calculus. Namely, if we prove that f, at $x > 1$ decreases with x for any fixed values of y and D within the relevant region of parameters, and take into account that $f(1,y,D) = 1$ for any acceptable values of y, D which is checked by substituting $x = 1$ into (S26), then we prove the inequality in question.

Calculate

$$\begin{aligned}\frac{\partial f}{\partial x} = {} & \frac{\log(y+1)\,(D\,y + D - 1)}{(D\,x\,y + D - 1)^2}\times \\ & \times\Big(\frac{(D-1)\,(x\,y+1)\,\log(x\,y+1) - x\,y\,(D\,x\,y + D - 1)}{(xy+1)(\log(x\,y+1))^2} + \frac{D-1}{\log D}\Big)\end{aligned} \tag{S27}$$

To determine the sign of $\partial f/\partial x$, one would have to concern oneself with the sign of the expression in the parentheses, which we rewrite as:

$$G(x,y,D) = \frac{D-1}{\log(x\,y+1)} - \frac{D\,x\,y + D - 1}{(\log(x\,y+1))^2} + \frac{D\,x\,y + D - 1}{(x\,y+1)(\log(x\,y+1))^2} + \frac{D-1}{\log D}, \tag{S28}$$

the only one of the factors constituting $\partial f/\partial x$ that has a chance to change its sign (all the others are positive under the conditions imposed by the situation under consideration). Introduce $z = x\,y + 1$ (note that, by the virtue of (S23), $z > 2$) and rewrite the expression (S28) in terms of z:

$$G(z,D) = \frac{D-1}{\log z} - \frac{Dz-1}{\log^2 z} + \frac{Dz-1}{z\,\log^2 z} + \frac{D-1}{\log D}. \tag{S29}$$

Differentiating (S29) in terms of z, we obtain

$$\frac{\partial G}{\partial z} = \frac{Dz-1}{z^2\,\log^3 z}\,(-z\,\log z + 2z - \log z - 2) \tag{S30}$$

Now one should concern oneself with the sign of the expression

$$g(z) = -z\,\log z + 2z - \log z - 2 \tag{S31}$$

Differentiate it twice in terms of z, to find

$$\frac{dg}{dz} = -\log z - \frac{1}{z} + 1 \tag{S32}$$

and

$$\frac{d^2g}{dz^2} = -\frac{1}{z} + \frac{1}{z^2}. \tag{S33}$$

Now d^2g/dz^2 turns zero when $z = 1$ and is positive at $z < 1$ and negative at $z > 1$, thus dg/dz has its maximum at $z = 1$. Calculating

$$\frac{dg}{dz}(1) = 0,$$

we see that at $z > 1$ the function $g(z)$ decreases and thus must be always less than $g(1) = 0$, so that $G(z)$, in its turn, decreases at $z > 1$.

Assume $z = 1 + \zeta$, $\zeta > 0$, $\zeta \ll 1$ and substitute in (S29). Upon expanding the Taylor series of $\log(1+\zeta)$ up to the required order of smallness, we get

$$G(1+\zeta) \simeq -\frac{D}{1+\zeta} + \frac{D-1}{\log D} < 0$$
$$\text{when } \zeta < \frac{D\,\log D - D + 1}{D-1} \tag{S34}$$

Thus at $z \to 1$ we have $G(z) < 0$ and, since $G(z)$ decreases with z when $z > 1$, we conclude that $G(z) < 0$ at $z > 1$. That is what makes $\partial f(x, y, D)/\partial x$ from (S27) negative in the area we are interested in, and thus $f(x, y, D)$ from (S26) at any fixed values y, D decreases at $x > 1$. Hence for any acceptable values of (x, y, D) we have $f(x, y, D) < f(1, y, D) = 1$ which proves (S26), and that, in its turn, is equivalent to having proved (S24).

Using elementary analysis we have proven that the inequality (S24) always holds and thus the range of g_2/g_1 fit for the coexistence always exists at any values of c_0/K_1 and $K_1/K_2 > 1$.

1.6 What are the coexistence conditions for two given species?

As we have already noted, there is a question more relevant in a laboratory: given two species and a resource, what should the initial concentration c_0 of this resource amount to, and which value of the dilution coefficient D should one take in order to get the bacteria steadily coexist in the serial dilution experiment?

Within the frames of our model, two species B_1, B_2 are given when their growth parameters (g_1, K_1) and (g_2, K_2) are known. We assume $g_1 > g_2$ and $g_1/g_2 < K_1/K_2$ (this being a necessary condition for the possibility of coexistence) and search for the values $c_0, D > 1$ for which the coexistence (mutual invasion) condition (S11) would be met.

Rewrite (S11) in terms of x, y, γ. As, again, $K_1 = x\,K_2$, $c_0 = y\,K_1 = x\,y\,K_2$ and $A = D\,c_0/(D-1) = D\,x\,y/(D-1)$, we get

$$\frac{(D\,x\,y + D - 1)\,\log D}{(D\,x\,y + x\,(D-1))\,\log D + (D-1)\,(x-1)\,\log(x\,y+1)} < \gamma < \frac{(D\,x\,y + D - 1)\,\log D - (D-1)\,(x-1)\,\log(y+1)}{(D\,x\,y + x\,(D-1))\,\log D} \tag{S35}$$

It is useful to remember that to enable coexistence one must have $c_0 > c^* > 0$, $c^* = (g_2\,K_1 - g_1\,K_2)/(g_1 - g_2)$ being the critical value of the initial concentration at which the "$K-$strategist" catches up with the "$r-$strategist" in terms of their growth rates. (Upon further depletion of the resource the "$K-$strategist" becomes the one that grows faster.) In terms of x, y, γ it's

$$y > y^* = \frac{\gamma x - 1}{x\,(1-\gamma)}. \tag{S36}$$

First it should be noted that at $D \to 1$ the leftmost side of the inequality (S35) tends to $\dfrac{xy}{xy + (x-1)\,\log(xy+1)}$. At $y \to \infty$ this amounts to 1. To see why, consider $\alpha(t) = (\log(t+1))/t$ at $t \to \infty$. Using the L'Hospital's rule at $t \to \infty$ we get $\alpha \to 0$. Dividing both the nominator and the denominator of l. h. s. of (S35) by xy and making y tend to ∞ we get $1/(1 + (x-1)\,\alpha(xy))$, which tends to 1 as $\alpha(xy) \to 0$.

Thus at $D \to 1$ and $y \to \infty$ we can always make l. h. s. of (S35) greater than γ, which is less than 1, say, at some $D = 1 + \epsilon$ (with $\epsilon > 0$) and $y = Y$.

On the other hand, at $y \to y^* = (\gamma x - 1)/(x\,(1-\gamma)$ and $D = xy + 1$ the l. h. s. of (S35) becomes

$$\frac{(D\,x\,y + D - 1)\,\log D}{(D\,x\,y + x\,(D-1))\,\log D + (D-1)\,(x-1)\,\log(x\,y+1)} = \frac{xy+2}{xy+2x} = \frac{\gamma\,x - 2\,\gamma + 1}{2x - 1 - \gamma\,x} \tag{S37}$$

Comparing this with γ is reduced to comparing 0 with $(\gamma\,x - 1)(1-\gamma) > 0$. Indeed, opening the parentheses in the latter (true under our assumptions) inequality we get $\gamma\,x - \gamma^2\,x - 1 + \gamma > 0$,

which is tantamount to $2\gamma\, x - \gamma - \gamma^2\, x > \gamma\, x - 2\gamma + 1$, i. e. $\gamma\,(2x - 1 - \gamma\, x) > \gamma\, x - 2\gamma + 1$, from which it follows that

$$\gamma > \frac{\gamma\, x - 2\gamma + 1}{2x - 1 - \gamma\, x}. \tag{S38}$$

Thus at $D = xy + 1$ and $y \to y^*$ (and thus at some $y = y^* + \delta$, $\delta > 0$) we get l. h. s. of (S35) less than γ.

A path in $(D, y)-$plane joining the points $(1 + \epsilon; Y)$ and $x\,(y^* + \delta) + 1; y^* + \delta)$ by the virtue of continuity will have a point $(D_0; y_0)$ at which l. h. s. $= \gamma$. As the r. h. s. of the same inequality (S35) is always greater than l. h. s., at that same point $(D_0; y_0)$ r. h. s. $> \gamma$. Somewhere in the close vicinity of $(D_0; y_0)$, again due to continuity, there exists a point $(D_1; y_1)$ at which l. h. s. $< \gamma$ and still r. h. s. $> \gamma$, so that γ can be indeed coaxed into the interval between l. h. s. and r. h. s. in some area of the $(D, y-)$plane. (Let us emphasize again that $\gamma = g_2/g_1$ is a fixed value in this subsection. We chose two strains or species B_1 and B_2 and look for the environmental parameters c_0, D that would ensure that the l. h. s. of (S35) is less than γ while at the same time r. h. s. of (S35) is greater than γ. While we cannot offer an analytical solution providing the pairs (c_0, D) thus favouring coexistence of B_1 and B_2, we can prove that such pairs do exist and offer a way to find them numerically.) This, however, is true only provided that the basic requirements $\gamma < 1, x\gamma = (K_1\, g_2)/(K_2\, g_1) > 1$ are satisfied.

To provide the conditions (c_0, D) favoring the coexistence of the bacteria B_1 and B_2 with the Monod parameters (g_1, K_1) and (g_2, K_2) respectively, we offer a code that inputs the Monod parameters and returns a plot picturing the area of (c_0, D) favouring coexistence (or the error message if the basic conditions mentioned above are not satisfied). All the points in the plot are clickable, so that the desirable numerical values can be obtained from it.

2 Coexistence in the case of "effectively finite" dilution period

In the previous section we have assumed the dilution period T "large enough" (for the whole initial amount of resource c_0 to be depleted during a dilution cycle), i. e. effectively infinite, and concluded that, for two strains or species $B_1\,(g_1, K_1)$ and $B_2\,(g_2, K_2)$ with $g_1 > g_2,\ g_1/g_2 < K_1/K_2$ within the frames of the Monod-driven dynamics in a serial dilution environment, the parameters c_0, D (the initial resource abundance and the dilution coefficient, respectively) favorable for the steady state coexistence always exist and can be numerically established. What if the dilution period is not "large enough", but rather noticeably finite, so that, during a dilution cycle, not all the resource gets depleted?

The change of outcome in this case cannot be ignored as it can happen that not just coexistence of two or more, but survival of any single species is endangered. Indeed, for a species $B\,(g, K)$ the growth rate through the whole dilution cycle cannot be greater than $g\, c_0/(c_0 + K)$, so that if $T < (c_0 + K)\, \log D/(g\, c_0)$, then towards the end of the said dilution cycle the species gets multiplied less than $D-$fold. Thus, from cycle to cycle, the culture will die out.

In this section, we address the following questions

- Given a dilution period T, what are the necessary and sufficient conditions for a single strain or species to survive?
- If there is a steady state for a monoculture, in which way is the final resource concentration c_f at the end of a dilution cycle connected to the dilution period T?
- For given c_0, D, K_1, K_2 and $T \to \infty$ there is the coexistence criterion (S11) establishing the upper and lower boundaries of the "coexistence region" of the ratio g_2/g_1 within which coexistence of the strains or species $B_1\,(g_1, K_1)$ and $B_2(g_2, K_2)$ is possible. What happens with that region when the dilution period T becomes smaller?
- Can the criterion (S11) be modified to account for the fact that not all the resource gets depleted during the dilution period?

- Given two strains or species that can coexist at $T \to \infty$ for given parameters c_0, D, can the critical value T_{cr} be established at which the coexistence becomes impossible?

Of the concerns listed above, that for the fate of the coexistence region from the criterion (S11) is, in a way, the easiest to address. Coexistence between species or strains B_1 (g_1, K_1) and B_2 (g_2, K_2) sharing the same resource is possible because through the first part of a dilution cycle B_1 multiplies faster, and through the second part the fastest of two is B_2. When the dilution period is less than (effective) infinity, B_2 has less resource to build upon during the phase of the cycle when it grows faster. Moreover, if the dilution period T is so short that, in the B_1-monoculture, the critical value $c^* = (g_2 K_1 - g_1 K_2)/(g_1 - g_2)$ is not reached, then B_2 has no chance to invade. On the other hand, a species with an exceptionally small $K-$constant that would have supplanted B_1 if the dilution cycle period were very large, in the case of smaller values of T may settle for coexistence because its comparative fitness would decrease. Thus, for given K_1, K_2 the coexistence region defined by (S11) would move to the right, favoring greater values of g_2/g_1. Moreover, it will become dependent on the absolute values of g_1, g_2 (to the point that species with too low maximum growth rates will not be able to survive even separately, much less to coexist), so that, for instance, the upper part of the banana-shaped area in Fig 1, **d** for given B_1 (g_1, K_1) will move down, the lower part will move up and get deformed in different ways for different pairs of values g_1, K_1.

The above (concerning the expected transformations of the coexistence region) will be further illustrated by the relevant formulae below.

2.1 Given the dilution period $T < \infty$, provided that the monoculture of a given strain survives, how much of the resource gets depleted through a dilution cycle? For a given strain or species, how large should be the dilution period to enable its survival as a monoculture?

Consider a strain or species $B\,(g, K)$ subject to serial dilution with the initial resource abundance c_0, dilution coefficient D and dilution period $T < \infty$. Assume that the system has reached its steady state and the monoculture B managed to survive. Within a dilution cycle, the dynamics goes as

$$\begin{aligned} \frac{dN}{dt} &= \frac{g\,c}{c+K}\,N, \\ \frac{dc}{dt} &= -\frac{g\,c}{c+K}\,N, \end{aligned} \tag{S39}$$

N, c being the current abundances of the species B and the resource respectively. If N_0 is its initial steady state abundance, then the conservation law that holds through each cycle has it

$$N_0 + c_0 = N_f + c_f = c^{(tot)} = \text{const}, \tag{S40}$$

N_f and c_f being the final abundances of the species B and the resource respectively.

In the steady state $N_f = DN_0$; combining this with (S40) we get

$$N_0 = \frac{c_0 - c_f}{D-1},\; c^{(tot)} = \frac{Dc_0 - c_f}{D-1}. \tag{S41}$$

Explore the connection between c_f and T. Using (S39) we get

$$T = \int_0^T dt = \int_{c_f}^{c_0} \frac{c+K}{g\,c\,(c^{(tot)} - c)}\,dc, \tag{S42}$$

which, upon integration and some further manipulations, reduces to

$$\frac{1}{g}\left(\log D + \frac{(D-1)\,K}{Dc_0 - c_f}\,\log\frac{Dc_0}{c_f}\right) = T \tag{S43}$$

This yields an implicit function $c_f\,(T)$; whenever the parameters are known, (S43) can be solved numerically.

Finally, at $c_f \to 0$ the dilution period T tends to infinity, and this was our case when we assumed nearly all the resource depleted at the end of each dilution cycle. At $c_f \to c_0$ we would have $T \to (c_0 + K)\,\log D/(g\,c_0)$. Due to the continuity of T as a decreasing function of c_f in (S42) we conclude that for each $T > (c_0 + K)\,\log D/(g\,c_0)$ there is a steady state with its own c_f, such that $0 < c_f < c_0$. As at $T \leq (c_0 + K)\,\log D/(g\,c_0)$ the microbe B cannot achieve $D-$fold multiplification, we also conclude that the necessary and sufficient condition for the monoculture $B\,(g, K)$ to survive at $T < \infty$ is indeed

$$T > \frac{(c_0 + K)\,\log D}{g\,c_0}. \tag{S44}$$

2.2 General (though implicit) form of the coexistence criterion when the dilution period $T < \infty$

Given two strains or species, $B_1\ (g_1, K_1)$ and $B_2\ (g_2, K_2)$, $g_1 > g_2$, $g_1/g_2 < K_1/K_2$, we assume that the dilution period T is at least large enough to allow each of the microbes grow $D-$fold as a monoculture.

$$T > \max\Big(\frac{c_0 + K_1}{g_1\,c_0}\,\log D\ ;\ \frac{c_0 + K_2}{g_2\,c_0}\,\log D\Big), \tag{S45}$$

It can be checked out that the condition (S45) in our case is equal to simply

$$T > \frac{c_0 + K_2}{g_2\,c_0}\,\log D. \tag{S46}$$

The coexistence (or mutual invasion) criterion is deduced in the same way as when the dilution period T is effectively infinite. To determine whether B_2 can invade the B_1-monoculture, we use

$$\frac{dc}{dt} = -\frac{g_1 c}{c + K_1}\,(c_1^{(tot)} - c),\ \text{with}\ c_1^{(tot)} = \frac{Dc_0 - c_{f1}}{D - 1}. \tag{S47}$$

The condition determining the invasion success of the microbe B_2 is

$$\int_0^T \frac{g_2\,c}{c + K_2}\,dt > \log D; \tag{S48}$$

we remind that, this way, a seed amount of B_2 in the environment shaped by B_1-monoculture will multiply more than $D-$fold through the dilution cycle and thus will be able to persist. Using (S47), the condition (S48) can be rewritten as

$$\int_{c_0}^{c_{f1}} \frac{g_2\,c}{c + K_2}\,\frac{dt}{dc}\,dc = \int_{c_0}^{c_{f1}} \frac{g_2\,c}{c + K_2}\Big(-\frac{c + K_1}{g_1\,c\,(c_1^{(tot)} - c)}\Big)\,dc = \frac{g_2}{g_1}\int_{c_{f1}}^{c_0} \frac{c + K_1}{(c + K_2)(c_1^{(tot)} - c)}\,dc > \log D. \tag{S49}$$

Performing the integration, the condition (S49) can be reduced to

$$\frac{g_2}{g_1}\Big(\frac{c_1^{(tot)} + K_1}{c_1^{(tot)} + K_2}\,\log D + \frac{K_1 - K_2}{c_1^{(tot)} + K_2}\,\log\frac{c_0 + K_2}{c_{f1} + K_2}\Big) > \log D \tag{S50}$$

Exchanging indices "1" for "2" and vice versa, we get the condition under which B_1 can invade the environment shaped by the monoculture B_2

$$\frac{g_1}{g_2}\Big(\frac{c_2^{(tot)} + K_2}{c_2^{(tot)} + K_1}\,\log D - \frac{K_1 - K_2}{c_2^{(tot)} + K_1}\,\log\frac{c_0 + K_1}{c_{f2} + K_1}\Big) > \log D \tag{S51}$$

Thus the modified coexistence criterion in the case of a finite dilution period reads as:

$$
\begin{aligned}
\frac{(c_1^{(tot)} + K_2)\,\log D}{(c_1^{(tot)} + K_1)\,\log D + (K_1 - K_2)\,\log \frac{c_0+K_2}{c_{f1}+K_2}} \;<\; \frac{g_2}{g_1} \;< \\
<\; \frac{(c_2^{(tot)} + K_2)\,\log D - (K_1 - K_2)\,\log \frac{c_0+K_1}{c_{f2}+K_1}}{(c_2^{(tot)} + K_1)\,\log D},
\end{aligned}
\tag{S52}
$$

with $c_{1,2}^{(tot)} = (Dc_0 - c_{f1,2})/(D-1)$ and $c_{f1,2}$ connected with the dilution period T in the way indicated by (S43). It is much less useful than the similar criterion in the case of the effectively infinite dilution period, as c_f is a value that depends in a complex way on the dilution period T **and** the constants g, K. Thus the boundaries in (S52), this time, are not explicit. Still, for any given values of the parameters c_0, D, g_1, K_1 and K_2 the inequality (S52) can be solved numerically in terms of g_2, so that, for all practical purposes, it is a workable criterion.

2.3 Given two bacterial strains or species B_1, B_2 able to coexist at $T \to \infty$ find the critical value of the dilution period T_{cr} at which coexistence disappears

In the limit $c_f \ll Dc_0$ (which is automatically the case when $D \gg 1$) it does not take calculations to see that both left l and right r boundaries in (S52) increase with $c_{f1,2}$ respectively and thus increase when the dilution period T gets shorter. Indeed, in this limit $c_1^{(tot)} = c_2^{(tot)} = D\,c_0/(D-1)$, so that dependence on $c_{f1,2}$ enters through logarithmic terms only. (By methods of elementary analysis it can be shown that both $l(T)$ and $r(T)$ decrease with T in any case, not just in this particular limit.)

Assume $B_1(g_1, K_1)$ and $B_2(g_2, K_2)$ such that $g_1 > g_2$, $g_1/g_2 < K_1/K_2$ coexist at $T \to \infty$, so that the criterion (S52) holds at $c_{f1,2} = 0$ (under these condition it reduces to (S11)). When the dilution period gets smaller, in the criterion (S52) which has the form $l(T) < g_2/g_1 < r(T)$ both boundaries $l(T)$ and $r(T)$ move to the right. Hence the left boundary $l(T)$ moves towards the value of the ratio g_2/g_1, and $r(T)$ moves from it. The coexistence becomes blocked at $T = T_{cr}$ such that $l(T_{cr}) = g_2/g_1$, or, in the limit $c_{f1} \ll D\,c_0$,

$$
\frac{(c^{(tot)} + K_2)\,\log D}{(c^{(tot)} + K_1)\,\log D + (K_1 - K_2)\,\log \frac{c_0+K_2}{c^*_{f1}+K_2}} \;=\; \frac{g_2}{g_1},
\tag{S53}
$$

with $c^*_{f1} = c_{f1}(T_{cr})$. Solving it for c^*_{f1} we get

$$
c^*_{f1} = -K_2 + (c_0 + K_2)\,D^{-\dfrac{(g_1 - g_2)\,c^{(tot)} + g_1\,K_2 - g_2\,K_1}{g_2\,(K_1 - K_2)}}
\tag{S54}
$$

Note that, in the limit $c_f \ll D\,c_0$, we should rewrite (S43) as

$$
\frac{1}{g}\Big(\log D + \frac{(D-1)\,K}{Dc_0}\,\log \frac{Dc_0}{c_f}\Big) = T,
\tag{S55}
$$

which gives us

$$
T_{cr} = \frac{1}{g_1}\Big(\log D + \frac{(D-1)\,K_1}{Dc_0}\,\log \frac{Dc_0}{-K_2 + (c_0 + K_2)\,D^{-\frac{(g_1-g_2)\,c^{(tot)} + g_1\,K_2 - g_2\,K_1}{g_2\,(K_1 - K_2)}}}\Big),
\tag{S56}
$$

T_{cr} being the value of the dilution period at which two given strains or species $B_1\,(g_1, K_1)$ and $B_2\,(g_2, K_2)$ able to coexist at $T \to \infty$ lose that ability. As $g_1 > g_2$, at $T > T_{cr}$ the strain or species B_1 comes out the sole winner of the serial dilution experiment.

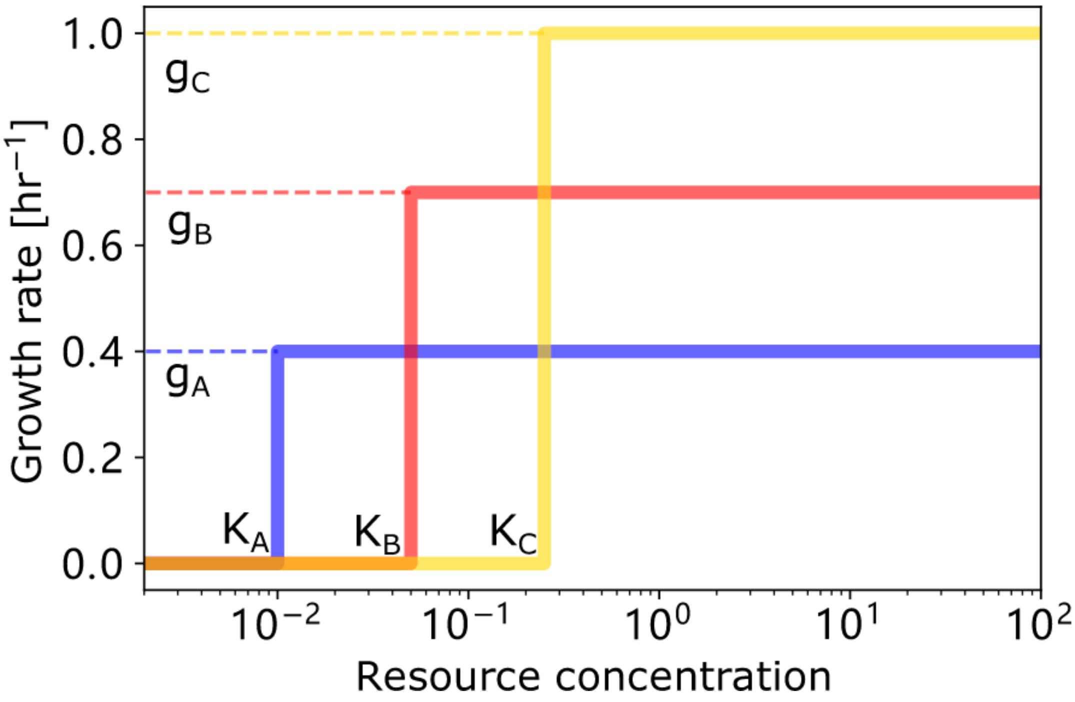

Figure C: **Growth curve of the simplified model.** The 3 solid lines show how growth rate depends on the resource concentration in our simplified model. Here g_A, g_B and g_C are approximations of the maximum growth rate from Monod's law of growth, and K_A, K_B and K_C are approximations of the substrate affinity.

3 Simplified model of Monod-like coexistence of multiple species on a single resource

3.1 Description of a model with the dynamics based on two parameters and allowing coexistence of any number of species on a single resource

The idea that the competitive exclusion principle might be violated in the serial dilution setting, once adopted, might play with the elementary algebraic associations one routinely exploits and thus has at hand. After all, the Monod's dynamics is characterised by two parameters (g, K); it might seem natural that it should, in principle, produce two solutions corresponding to two survivors supported by a single shared resource. In fact, this kind of intuition here goes amiss. In principle, we could have any number of survivors on a single resource. (The range of parameters favoring such coexistence grows smaller with each new prospective survivor added, so that, in reality, it is almost impossible to arrange the coexistence of more than two or three species.)

To see why that happens, consider a simple Monod-inspired model. Let B_i be a microbe species that grows exponentially on a resource c with the maximum growth rate g_i as long as the resource concentration $c > K_i$. Then it stops growing. However discontinuous, that would still be dynamics that is characterised by two parameters g_i, K_i. Fig (S3) shows three species and their growth rates as dependent on the resource concentration c.

We consider the same serial dilution setting as before, with the dilution coefficient D and the length of the dilution cycle long enough for the bacteria to consume as much of the resource as they can.

Just as in the case with the real Monod's dynamics, it is easy to see that two microbes B_1, B_2 can coexist using the same single resource c, as long as, if $K_1 > K_2$, then also $g_1 > g_2$ (otherwise one microbe at any point will grow faster than the other, until both stop growing, and thus

will become the sole winner). Indeed, in the steady state, when the point $c = K_1$ is reached, the concentration N_1 of the microbe B_1 should grow $D-$fold compared to the initial concentration $N_{1,0}$ at the beginning of a dilution cycle within the possibilities granted by the exponential growth, $N_1 = DN_{1,0}$. Thus this should happen at the time point $T_1 = (\log D)/g_1$. By that time the microbe B_2 growing exponentially with the rate g_2 will have the concentration $N_2 = D^{g_2/g_1} N_{2,0}$, where $N_{2,0}$ is the initial concentration of the microbe B_2 at the beginning of a dilution cycle in the steady state. The biomass conservation law at this point will read as

$$K_1 + D\,N_{1,0} + D^{\frac{g_2}{g_1}}\,N_{2,0} = c_0 + N_{1,0} + N_{2,0}, \tag{S57}$$

c_0 being the initial concentration of the resource at the beginning of a dilution cycle (, it should be greater than K_1).

Now B_1 stops growing and B_2 continues growing exponentially till the point $c = K_2$. From that time point till the end of the dilution cycle the biomass conservation law reads simply as

$$K_2 + DN_{1,0} + DN_{2,0} = c_0 + N_{1,0} + N_{2,0} \tag{S58}$$

From (S58) we get, understandably, $N_{1,0} + N_{2,0} = \dfrac{c_0 - K_2}{D-1}$, so that if coexistence indeed takes place, each of the steady state concentrations of both species should lie between 0 and $\dfrac{c_0 - K_2}{D-1}$. Using this consideration, from (S57) and (S58) we get

$$0 < N_{2,0} = \frac{K_1 - K_2}{D - D^{\frac{g_2}{g_1}}} < \frac{c_0 - K_2}{D-1}, \tag{S59}$$

In the limit $c_0 >> K_2$ the coexistence condition takes the form

$$K_1 - \frac{D - D^{\frac{g_2}{g_1}}}{D-1}\,c_0 < K_2 < K_1. \tag{S60}$$

This is a fairly mild restriction, as a coexistence condition. For instance, if $g_2 \to 0$, the bacterium B_2 will be able to invade for any $K_2 < K_1$ (though it may be present in a very small amount if K_2 is close to K_1). This is because at $c = K_1$ the microbe B_1 stops growing, and the biomass equal to $K_1 - K_2$ is always granted to B_2. In fact, it is more restrictive for B_1 that grows faster, but has larger $K-$constant. Real Monod's dynamics does not work this way: the strain or species with greater maximum growth rate does not stop growing when the resource abundance equals its affinity K, as at this point its growth rate is only reduced by half compared to maximum. So at this point it definitely stays in the game. Yet there are similarities, especially when the $K-$constants of both competitors are very different.

Suppose there are three microbes trying to share the same environment containing the single source c, under the same serial dilution conditions; for the sake of definiteness, we assume $g_1 > g_2 > g_3$ and thus, to make coexistence possible, it should also happen that $K_1 > K_2 > K_3$. If they succeed in sharing, then, at the steady state, the time point at which B_1 stops growing must be $T_1 = \log D/g_1$ (and at that time we should have $c\,(T_1) = K_1$; the microbe B_2 stops growing must be $T_2 = \log D/g_2$ (at $c\,(T_2) = K_2$) and, finally, B_3 stops growing at the time point $T_3 = \log D/g_3$ (at $c\,(T_3) = K_3$). Thus, instead of (S57) and (S58) we will have the following system of equations describing the biomass conservation at each of these three points:

$$\begin{cases} (D-1)\,N_{1,0} + (D^{\frac{g_2}{g_1}} - 1)\,N_{2,0} + (D^{\frac{g_3}{g_1}} - 1)\,N_{3,0} = c_0 - K_1 \\ (D-1)\,(N_{1,0} + N_{2,0}) + (D^{\frac{g_3}{g_2}} - 1)\,N_{3,0} = c_0 - K_2 \\ (D-1)\,(N_{1,0} + N_{2,0} + N_{3,0}) = c_0 - K_3 \end{cases} \tag{S61}$$

To begin with, it is realizable, meaning that there exists a set of parameters $(g, K), c, D$ that would ensure such coexistence. Take any set $g_1 > g_2 > g_3$, $D > 1$, and for any positive numbers $N_{1,0}, N_{2,0}, N_{3,0}$ the values $c_0 - K_1$, $c_0 - K_2$ and $c_0 - K_3$ will be obtained. Fix any $c_0 > (D -$

$1)\,(N_{1,\,0} + N_{2,\,0} + N_{3,\,0})$ and get $K_1, K_2, K_3 < c_0$ ordered as required. Next, in this model, it is even quite realizable. Solving the system (S61) we get for B_3

$$N_{3,\,0} = \frac{K_2 - K_3}{D - D^{\frac{g_3}{g_2}}} > 0 \tag{S62}$$

,

then for B_2

$$N_{2,\,0} = \frac{(D - D^{\frac{g_3}{g_2}})\,K_1 - (D - D^{\frac{g_3}{g_1}})\,K_2 + (D^{\frac{g_3}{g_2}} - D^{\frac{g_3}{g_1}})\,K_3}{(D - D^{\frac{g_3}{g_2}})\,(D - D^{\frac{g_2}{g_1}})} > 0 \tag{S63}$$

,

and, finally, for B_1

$$\begin{aligned} N_{1,\,0} = {} & \frac{c_0 - K_3}{D - 1} - \\ & - \frac{(D - D^{\frac{g_3}{g_2}})\,K_1 - (D - D^{\frac{g_3}{g_1}})\,K_2 + (D^{\frac{g_3}{g_2}} - D^{\frac{g_3}{g_1}})\,K_3}{(D - D^{\frac{g_3}{g_2}})\,(D - D^{\frac{g_2}{g_1}})} - \\ & - \frac{K_2 - K_3}{D - D^{\frac{g_3}{g_2}}} > 0. \end{aligned} \tag{S64}$$

It can be seen from above that the conditions are still fairly easy to satisfy if c_0, K_1, K_2 and K_3 are well separated.

Coexistence of n microbes $B_1, B_2, ..., B_n$ is governed by the system of equations

$$\begin{cases} (D-1)\,N_{1,\,0} + (D^{\frac{g_2}{g_1}} - 1)\,N_{2,\,0} + (D^{\frac{g_3}{g_1}} - 1)\,N_{3,\,0} + ... + (D^{\frac{g_n}{g_1}} - 1)\,N_{n,0} = c_0 - K_1 \\ (D-1)\,(N_{1,\,0} + N_{2,\,0}) + (D^{\frac{g_3}{g_2}} - 1)\,N_{3,\,0} + ... + (D^{\frac{g_n}{g_2}} - 1)\,N_{n,0} = c_0 - K_2 \\ ... \\ (D-1)\,(N_{1,\,0} + N_{2,\,0} + N_{3,\,0} + ... + N_{n,0}) = c_0 - K_n. \end{cases} \tag{S65}$$

As with (S61), it is easy to see that such a coexistence is realizable. Take any set of the parameters $g_1, ..., g_n$, any $D > 1$ and any set of positive $N_{1,\,0}, ..., N_{n,0}$, assume any $c_0 > (D-1)\,(N_{1,\,0} + N_{2,\,0} + N_{3,\,0} + ... + N_{n,0})$ and get $K_1, ..., K_n$, again automatically ordered as required.

In this model, any new survivor's survival is relatively likely, and indeed we get a large number of microbes surviving on a single source in our simulations. It is not so with the Monod's dynamics, with which it is hard to get two species coexisting, much harder yet to get three and almost impossible further on. Nevertheless, in principle, the parameters can be found to get such a coexistence. To see why it should happen, break the Monod's curve into a sequence of flat Heaviside's steps, so that it is approximated by a "stairs", each of the steps corresponding to a new value of the growth rate. In this way, we look at the single resource c as a set of a number of unique resources, each providing its own value of the (exponential) growth rate for each microbe. Indeed, any monotonically increasing growth rate function may be approximated this way, by an arbitrary number of resources and steps. In the study, ref. [2] we show that, in principle, in a serial dilution setting it is possible in this case to have the number of survivors match the number of resource. However, it works best when the microbes have different resource preferences: say, one of them starts consuming resource number 2 first, while the other one starts with the resource number 1 etc. In our case, species are bound to have the same resource preferences, and in this case multiple coexistence is much harder (though possible) to arrange.

Note that, even if breaking the Monod's curve in any number of steps we could hope for any number of survivors, we play on a shaky ground. As the range of the parameters favoring the coexistence of more and more species becomes smaller, we can well fall out of the accuracy limits of the Monod's dynamics. Being in itself an approximation, it cannot support that sort of handling to the infinity.

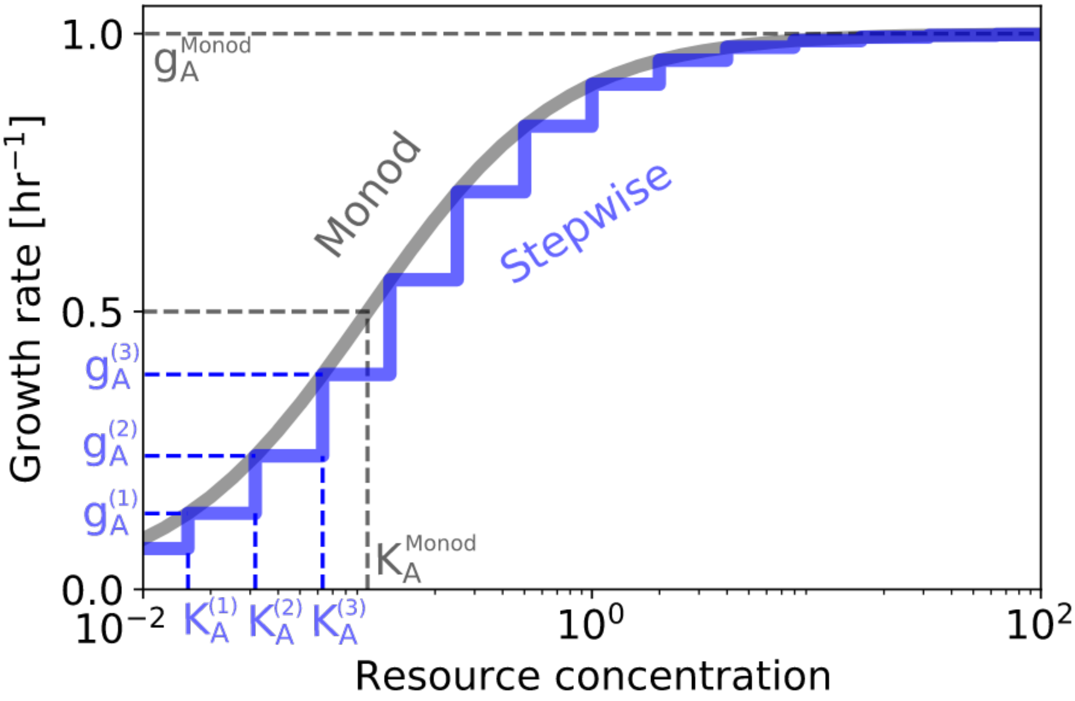

Figure D: **Monod growth curve approximated by the simplified model.** The grey line shows the growth curve of a strain that obeys Monod's law of growth on a single resource, with maximum growth rate g_A^{Monod} and affinity K_A^{Monod}. The blue line is the growth growth curve of a strain that grows on a set of unique resources and follows our simplified growth model with growth rates $g_A^{(1)}, g_A^{(2)}$... etc. and affinity $K_A^{(1)}, K_A^{(2)}$... etc.

3.2 Phase volume and probability scaling for coexistence of n different strains or species on a single resource

Here we concern ourselves with the following question: within the frames of the simplified model, how hard is it for a new species to invade the environment shared by a certain number of strains or species, each with its unique parameters (g_i, K_i), and, instead of supplanting any of them, rather join the coexisting community? In the other words, how does the probability of n randomly chosen strains or species to coexist depend on their number n?

To address this question, rewrite (S57) and (S58) describing the steady state in which two strains or species $B_1(g_1, K_1)$ and $B_2(g_2, K_2)$ coexist through an infinite number of dilution cycles, in vector form:

$$N_{1,0}\begin{pmatrix} D-1 \\ D-1 \end{pmatrix} + N_{2,0}\begin{pmatrix} D^{\frac{g_2}{g_1}}-1 \\ D-1 \end{pmatrix} = \begin{pmatrix} c_0 - K_1 \\ c_0 - K_2 \end{pmatrix} \tag{S66}$$

As it has been noted in the previous subsection, assigning various positive numbers to $N_{1,0}$ and $N_{2,0}$ we span the region of possible values of $c_0 - K_1$, $c_0 - K_2$ allowing for coexistence of these two strains or species, the necessary condition for coexistence ($K_1 > K_2$ given that $g_1 > g_2$) being satisfied automatically. Intuition suggests that, for given g_1, g_2 of the pair of microbes whose coexistence is in question, the ratio of the area of the region allowing for coexistence to that of the whole compact $K-$parameter space must characterize the probability of the event in which randomly chosen constants K_1, K_2 will allow for coexistence. To make it more precise, consider a vector space pertaining specifically to a serial dilution process. The first axis of the coordinate space associated with it will give the bacterial abundances at the point $c = K_1$, when the first strain or species stops growing. The second axis is that of bacterial abundances at the point $c = K_2$, when the second strain also stops growing. The units pertaining to these axes (designated further as $\mathbf{1}$) are assumed to be some relevant quanta of abundance, so that a point marked $D-1$ or $D^{g_2/g_1}-1$ in Fig (S5) are, in fact, $(D-1)\times\mathbf{1}$ and $(D^{g_2/g_1}-1)\times\mathbf{1}$, while the coordinates' values c_0 or $c_0 - K_{1,2}$ are supposed to be expressed in those same units. Thus (see Fig (S5)) vector $\vec{B_1}\Big((D-1)\times\mathbf{1},\,(D-1)\times\mathbf{1}\Big)$ represents the strain or species B_1 in the steady state, while vector $\vec{B_2}\Big((D^{g_2/g_1}-1)\times\mathbf{1},\,(D-1)\times\mathbf{1}\Big)$ represents the strain or species B_2. Any positive linear combination of the vectors $\vec{B_1}, \vec{B_2}$, represented by a point in the blue-shaded area (or, to be exact, by its radius-vector) in Fig (S5), corresponds to a pair of values K_1, K_2 that would allow for coexistence under given external conditions. For an arbitrary distribution of the half-growth parameter K, the phase volume occupied by the points $(c_0 - K_1, c_0 - K_2)$ favoring coexistence is the (weighted) area of the blue-shaded region, with each pixel coming with its own probability density, and the probability for two strains or species with given growth rates $g_1, g_2 < g_1$ and randomly chosen K_1, K_2 to coexist is given by the ratio of the area of the blue-shaded region to the (weighted) area of the whole quadrant with the side c_0. If the parameter K is **uniformly** distributed, then we deal with the ratio of the geometric areas, which amounts to

$$P(g_1, g_2) = \frac{1}{2!}\det\begin{pmatrix} D-1 & D^{\frac{g_2}{g_1}}-1 \\ D-1 & D-1 \end{pmatrix}\frac{1}{(D-1)^2} = \frac{1}{2}\,\frac{D-D^{\frac{g_2}{g_1}}}{D-1}. \tag{S67}$$

If the growth rates are also uniformly distributed, say, within an interval $(0; G)$, G being the ultimately maximum growth rate possible for a microbe, the probability for the event in which two randomly chosen strains or species $B_1(g_1, K_1)$ and $B_2(g_2, K_2)$, $g_2 < g_1$, can coexist in the given environment can be calculated as

$$P_2 = \int_0^G\int_0^{g_1} P(g_1, g_2)\,dg_2\,dg_1 = \int_0^G\int_0^{g_1}\frac{1}{2}\,\frac{D-D^{\frac{g_2}{g_1}}}{D-1}\,dg_2\,dg_1. \tag{S68}$$

Calculate

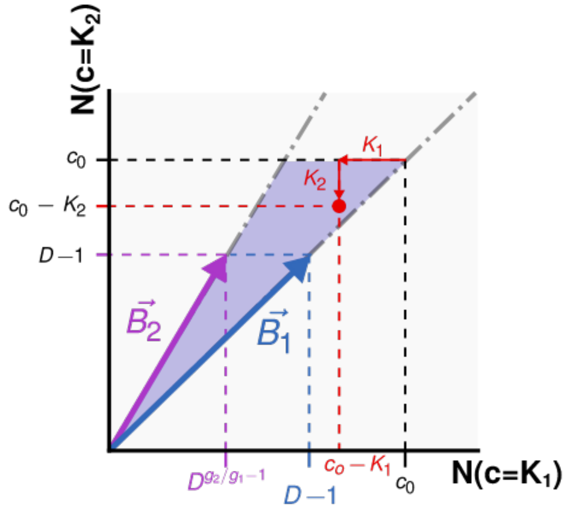

Figure E: **2-dimensional vector space representing steady state of a serial dilution process.** Horizontal axis serves to measure the species abundances at the point $c = K_1$ while vertical axis measures species abundance at the point $c = K_2$ scaled to some appropriate unit of abundance. Vectors $\vec{B_1}$ and $\vec{B_2}$ represent species B_1 and B_2 respectively, their coordinates proportional to the abundances of these species at the points $c = K_1$ (first coordinate) and $c = K_2$ (second coordinate) respectively. The blue-shaded area consists of points representing positive linear combinations of vectors $\vec{B_1}, \vec{B_2}$. A red point within the blue-shaded area, with the parameters K_1 and K_2 corresponding to it (shown in red color) provides the values K_1, K_2 favoring coexistence of two species with given growth rates g_1, g_2 in the given environment (as any other point in the blue-shaded area does). Coordinates of vectors $\vec{B_1}$, $\vec{B_2}$ given in dimensionless numbers are to be interpreted as number×unit of abundance

$$\int_0^G \int_0^{g_1} D^{\frac{g_2}{g_1}} \, dg_2 \, dg_1 = \int_0^G \int_0^{g_1} \exp\left((\log D) \frac{g_2}{g_1} \right) dg_2 \, dg_1 =$$

$$= \int_0^G \left(\frac{g_1}{\log D} \exp\left(\frac{g_2}{g_1} \log D \right) \Big|_0^{g_1} \right) dg_1 = \int_0^G \frac{g_1}{\log D} (D-1) \, dg_1 = \frac{G^2 (D-1)}{2 \log D}. \tag{S69}$$

As the whole region with $g_1 > g_2$ has the area $G^2/2$,

$$P_2 = \frac{1}{2} \left(\frac{D}{D-1} - \frac{1}{\log D} \right) \tag{S70}$$

(The same result would have been easier obtained if one had noted that within the interval $(0; g_1)$ (meaning that g_1 is fixed and we deal with a conditional probability) the ratio g_2/g_1 is in fact uniformly distributed. However, we have opted for demonstrating the more general procedure.) For large D the probability in (S70) is close to 1/2, yet another reminder that the simplified model is not a true reflection of (Monod's) reality. Still, it might provide some notion of the way the scaling should go with the number of coexisting species growing (see below).

In the case of three microbes, with the steady state coexistence conditions given by (S61), the similar considerations, this time in a 3-d vector space, allow one to obtain the probability for three strains of species with given $g_1 > g_2 > g_3$ and randomly chosen K_1, K_2, K_3, all $K_i < c_0$, to coexist:

$$P(g_1,g_2,g_3)=\frac{1}{3!}\det\begin{pmatrix}D-1 & D^{\frac{g_2}{g_1}}-1 & D^{\frac{g_3}{g_1}}-1\\ D-1 & D-1 & D^{\frac{g_3}{g_2}}-1\\ D-1 & D-1 & D-1\end{pmatrix}\frac{1}{(D-1)^3}=$$
$$=\frac{1}{3!}\det\begin{pmatrix}D-D^{\frac{g_2}{g_1}} & D^{\frac{g_2}{g_1}}-D^{\frac{g_3}{g_1}} & D^{\frac{g_3}{g_1}}-1\\ 0 & D-D^{\frac{g_3}{g_2}} & D^{\frac{g_3}{g_2}}-1\\ 0 & 0 & D-1\end{pmatrix}\frac{1}{(D-1)^3}= \tag{S71}$$
$$=\frac{1}{3!}\frac{(D-D^{\frac{g_2}{g_1}})(D-D^{\frac{g_3}{g_2}})}{(D-1)^2}.$$

Now the ratios g_2/g_1 and g_3/g_2 are, as random values, independent of each other, hence (in the case of uniform distribution of all the parameters g_i, K_i) the probability P_3 for three microbes with randomly chosen parameters to coexist can be calculated as the product of the factors structurally similar to P_2 in (S70), so that

$$P_3=\frac{1}{3!}\Big(\frac{D}{D-1}-\frac{1}{\log D}\Big)^2 \tag{S72}$$

Considering the case of n species, coexistence steady state given by (S65), upon similar calculations we get the probability P_n

$$P_n=\frac{1}{n!}\,q^{n-1}, \tag{S73}$$

with

$$q=\Big(\frac{D}{D-1}-\frac{1}{\log D}\Big). \tag{S74}$$

Let it be emphasised that the result (S73) is obtained within the frames of the simplified model under the assumption that both K and g parameters of the species are uniformly distributed. In reality the probability of n strains or species to coexist on a single source is expected to decrease with the number n even faster.

4 Coarse-graining and chemostat limit

If we were to coarse-grain our serial dilution picture, say, by plotting average concentration of a microbe across each cycle against time, with the time scale unit very large compared to the dilution period, we would get a curve tending to a constant line. Throughout this work we discussed invasion events occurring in the system that has already reached its steady state (corresponding to points of the above-mentioned constant line). It is tempting to consider the limit in which serial dilution curves are coarse-grained to reveal dynamics similar to the continuous dynamics of the cultures that grow in a chemostat. In this limit however coexistence of multiple strains or species on a single source should disappear, and we should be able to show that it happens in this limit in our model.

For a monoculture growing in a chemostat, the dynamics is driven by

$$\begin{aligned}\frac{dN}{dt}&=\frac{gc}{c+K}N-\delta N\\ \frac{dc}{dt}&=\Phi-\frac{gc}{c+K}N-\delta c,\end{aligned} \tag{S75}$$

where δ is the chemostat dilution rate and Φ the (constant) nutrient flux delivered to the environment.

Placing this in parallel to a serial dilution experiment, we would like to coarse-grain the latter in such a way that differentiating over time would be equivalent to dividing the increment of a

value (say, microbe or resource abundance) gained over the dilution period T by the length of the dilution period, so that for any relevant function $f(t)$ its derivative over time may be approximated as $df/dt \simeq (f(t+T) - f(t))/T$. We would also need to match the continuous dilution rate δ with the parameter of discrete acts of dilution, the dilution coefficient D, in the unique way that would allow to make serial dilution to chemostat transition.

Rewrite the first equation of (S75) as

$$\frac{N(t+T) - N(t)}{T} = \frac{g\,c(t)}{c(t)+K}\,N(t) - \delta\,N(t). \tag{S76}$$

There are two terms in the r. h. s. of (S76), the first one responsible for growth and the second one due to dilution. We do have those in serial dilution, even if growth happens first and dilution later; the "sampled", discrete form of the differential equation (S76) allows one to interpret the first term in its r. h. s. as growth during a dilution cycle and the second term as the effect of consequent dilution at the end of the cycle. Note that the coarse-graining, if correctly accomplished, should adequately reflect the steady state as well as all the previous progress leading to it. In the steady state the initial abundance of a surviving species N_0 at the end of cycle growth $D-$fold and then gets diluted to the point where it becomes again N_0. Looking for the right way to make a transition from serial dilution to chemostat dynamics, we can write for the steady state

$$\frac{N(t+T) - N(t)}{T} = \frac{N(t+T) - N(t+T-0) + N(t+T-0) - N(t)}{T}, \tag{S77}$$

where $N(t+T-0)$ should be interpreted as the species abundance at the end of the dilution cycle, so that $N(t+T-0) = DN_0$, and $N(t+T) = N(t) = N_0$. Thus we can separate the two terms suggested by (S76) according to their role both in chemostat and serial dilution dynamics:

$$\begin{aligned} \frac{N(t+T-0) - N(t)}{T} &= \frac{DN_0 - N_0}{T} = \frac{gc_0}{c_0+K}\,N_0 \\ \frac{N(t+T) - N(t+T-0)}{T} &= \frac{N_0 - DN_0}{T} = -\delta N_0 \end{aligned} \tag{S78}$$

We map parameters of discrete dynamics to those of continuos dynamics according to the following rules:

$$\begin{aligned} D - 1 &= \frac{gc_0}{c_0+K}\,T \\ D - 1 &= \delta\,T \end{aligned} \tag{S79}$$

(Note that, by virtue of (S79), to have the same chemostat representation of their monoculture serial dilution dynamics, two strains or species $B_1(g_1, K_1)$ and $B_2(g_2, K_2)$ should satisfy $g_1c_0/(c_0 + K_1) = g_2c_0(c_0 + K_2) = \delta$, that is, the same degeneration (specific functional dependence) in $g, K-$parameters required to coexist in chemostat where c_0 is interpreted as the steady state resource abundance.)

It remains to point out that, in fact, to get a good chemostat representation, serial dulution setting should satisfy even stricter constraints. In the case of chemostat with constant flux of the nutrient and constant dilution rate, the abundance of a species does not change in the steady state. To be able to coarse-grain our serial dilution experiment to resemble the chemostat dynamics, we should assume $D - 1 \ll 1$, unless we are willing to accept the situation in which the steady state values of N and c are not well-defined. (Indeed, checking the value of, say, species abundance N in the beginning of the dilution cycle we get N_0 and towards the end of the cycle — DN_0, and at other moments we get anything in between; if all those values are not close to each other, there is no steady state in terms of continuous dynamics.) So the coarse-grained steady state dynamics of serial dilution can be reduced to the continuous steady state dynamics (or should we say statics) of chemostat in the limit $D - 1 \to 1$.

If a strain or species $B_1(g_1, K_1)$ is subject to a serial dilution experiment with dilution coefficient $D = 1+\epsilon$, with $\epsilon \ll 1$, and dilution period $T = \epsilon\,\dfrac{c_0 + K_1}{g_1\,c_0}$ to match (S79), another strain or species

$B_2(g_2, K_2)$ with $g_2 < g_1$, to be able to coexist with B_1, should at least be able to survive in this environment. The necessary (though by no means sufficient) condition would be

$$\frac{g_2\, c_0}{c_0 + K_2} > \frac{\log D}{T}. \tag{S80}$$

If the above doesn't hold, the strain or species B_2 will grow through a dilution period less than $D-$fold and thus will never be able to survive. Substituting $\log D \simeq \epsilon - \epsilon^2/2$ and $T = \epsilon\,(c_0 + K_1)/(g_1 c_0)$ we get

$$\frac{g_2}{g_1} > (1 - \frac{\epsilon}{2})\,\frac{c_0 + K_2}{c_0 + K_1} \tag{S81}$$

Note that

$$\frac{c_0 + K_2}{c_0 + K_1} - \frac{g_2}{g_1} = \frac{(g_1 - g_2)\,c_0 - (g_2\,K_1 - g_1\,K_2)}{(c_0 + K_1)\,g_1} > 0, \tag{S82}$$

as $B_1(g_1, K_1)$ and $B_2(g_2, K_2)$, $g_1 > g_2$ are assumed to be able to coexist at some value of D, and this implies $g_1/g_2 < K_1/K_2$ and also $c_0 > c^* = (g_2\,K_1 - g_1\,K_2)/(g_1 - g_2)$. (It is useful to remember that c^* is the critical value of the resource abundance at which B_1 starts growing slower than B_2.) Thus from (S81) and (S82) we get

$$(1 - \frac{\epsilon}{2})\,\frac{c_0 + K_2}{c_0 + K_1} < \frac{g_2}{g_1} < \frac{c_0 + K_2}{c_0 + K_1} \tag{S83}$$

Thus the width of the region of values of $\frac{g_2}{g_1}$ with given K_1, K_2 goes to zero with $\epsilon \to 0$. Hence for any two randomly chosen strains or species, even if they are able to coexist under some conditions, coexistence becomes an event with zero probability at $\epsilon \to 0$.

5 Properties of a community of survivors on a single resource

In this section, the following questions are addressed:

- Suppose a community of strains or species is found surviving on a single resource due to feast-famine cycles. What can be predicted regarding the growth parameters g, K of survivors?
- Given a large pool of strains or species with their growth parameters g, K that can repeatedly invade the environment, suggest an algorithm effectively narrowing the set of potential survivors down to one or a few to facilitate subsequent simulations.

Any two strains or species that share the same resource supplied in a boom and bust manner must each have to itself a time period in which it grows faster than its counterpart. Otherwise, if through all the growth time one of them is always faster than the other, the slower strain or species would be driven out of the environment. For the Monod-abiding dynamics, that means that if $g_i > g_j$, than $g_i/K_i < g_j/K_j$, as we have shown in the previous Supplementary sections. Hence such a community would demonstrate the following pattern: if all the growth rates of the strains or species present are ordered as

$$g_1 > g_2 > \cdots > g_n, \tag{S84}$$

then one should have

$$K_1 > K_2 > \cdots > K_n, \tag{S85}$$

and also, somewhat less trivial,

$$g_1/K_1 < g_2/K_2 < \cdots < g_n/K_n. \tag{S86}$$

One will note that (S85) follows from (S84) and (S86) taken simultaneously.

If such a pattern is not observed, than either the growth dynamics of the species in the community does not respect Monod's law or the coexistence in question is transient and the steady state is yet to be reached.

Now to picking up survivors out of a large pool of competitors for a single resource.

Given a large pool of strains or species capable of invading the same environment containing a single nutrient within the frames of a serial dilution experiment, their growth parameters known and listed, the strains and species found in the steady state stable with respect to invasions, in the general case, should be found by simulation. However the capacities required to perform one can be significantly reduced. Below we assume that the initial resource abundance c_0 is large enough, larger than the critical value of the resource abundance for each pair of competitors. If it isn't the case, the algorithm is corrected to account for this condition. (We remind the reader that the critical resource abundance of a pair of strains or species is the abundance at which their growth rates equal each other.) (1). It would be wise to start with ordering. Namely, take the strain or species with the largest value of g, assign to it number 1, so it is $B_1(g_1, K_1)$ and strike out all the strains of species with lesser growth rate and greater half-growth constant K. Those would not be able to survive in the presence of B_1. Take the second-best in terms of growth rate alone among survivors and repeat the process (there is no need to include B_1 in this step) and so on, until all the strains or species left are ordered as $g_1 < g_2 < \cdots < g_n$ and at the same time $K_1 < K_2 < \cdots < K_n$. (2). Carry the ordering to a higher degree of sophistication: if there are two strains or species $B_i(g_i, K_i)$ and $B_j(g_j, K_j)$ with $g_i > g_j$ and also $g_i/K_i > g_j/K_j$, then B_j loses to B_i at any time point of the dilution period and should be stricken out. This is because the critical value of the resource abundance at which B_j starts growing faster than B_i, $c^* = (g_2 K_1 - g_1 K_2)/(g_1 - g_2)$, should be positive for B_j to have a chance. (3) When the pool is (hopefully) sufficiently reduced and renumeration achieved, it's time to start simulation. In one decides to opt for simulating subsequent invasion of each species instead of simultaneous invasion of all, it may be wise to let each species invade a number of times.

References

1. Lechmann L, Mullon C, Akcay E, Van Cleve J. Invasion fitness, inclusive fitness, and reproductive numbers in heterogeneous populations. Evolution. 2016;70(8):1689–1702.

2. Wang Z, Goyal A, Dubinkina V, George AB, Wang T, Fridman Y, et al. Complementary resource preferences spontaneously emerge in diauxic microbial communities. Nature communications. 2021;12(1):1–12.
