# Supplementary material for: Fine-scale diversity of microbial communities due to satellite niches in boom and bust environments
Source: PLoS Comput Biol. 2022 Dec 27;18(12):e1010244. doi: 10.1371/journal.pcbi.1010244 (PMC9829172; doi:10.1371/journal.pcbi.1010244)
Supplement: Attachment — Submitted filename: Review_comments.pdf [file pcbi.1010244.s003.pdf]

In their manuscript “Fine-scale diversity of microbial communities due to satellite niches in boom-and-bust environments”, Wang et al. examine the role of temporal niches and R-K like tradeoff in sustaining fine-scale diversity using simple models. The manuscript is well written and is addressing an interesting question. The models are simple but illustrate the general point the authors wish to make. I think the manuscript makes a timely point that is of interest to the community studying microbial ecology and for this reason is potentially well-suited for publication in PLoS Comp Bio.

However, I do feel that the manuscript is currently quite minimal. It is a good starting point, but I would like to see a number of points addressed more systematically. Currently, the models essentially reproduce intuitions first derived using graphical arguments by Levins in the context of Monod kinetics. The derivations are solid and calculations are correct. I especially like the arguments in section SI 1.4. A strong point of the current approach is that one can understand the coexistence region mechanistically at least in these simple settings. However, there are a lot of specialized assumptions made.

1. Please move S3 to main text. Otherwise, it is harder to follow. I would like to understand what happens when the growth rate (dN/dt) and the impact vector (dc/dt) are not exactly identical with opposite sign. We know that from the chemostat case that this assumption corresponds to the existence of a minimization principle. In general, we expect these to be correlated but not exact. Technically, the conservation law in S4 will be much more complicated and it would be interesting to at least understand when does the coexistence region get bigger or smaller.

2. Another interesting question is what happens if we no longer allow all the resources to be depleted and instead change time-scale of booms? How does co-existence region change? Technically, this seems to be assumed in derivation leading to S6.

3. In Equation S12, I am not sure what the small parameter is. I think it is 1/D. However, I cannot tell if other approximations are also being made (do I have to assume zeroeth order in K/c_o as well?). I would like to see corrections to next order in 1/D to better understand this. I am always a little confused what is held fixed and what is changing in the perturbations. This should not be too hard but may add some intuition to the problem.

4. Is there a way to derive a general scaling for the volume of the co-existence region as a function of number of species that need to co-exist. It seems like this might be possible from SI section 2. I tried and it was not super obvious to me but the authors clearly have thought about the problem much more than me.

Minor Points.

1. In deriving S6, would be useful to say assume all resources are always depleted to derive.

2. It would be helpful in derivation of S8-S10 to make connection to the idea of invasion fitness in static literature that the idea is to find when both species can invade each other at small numbers.
