# Supplementary material for: Fine-scale diversity of microbial communities due to satellite niches in boom and bust environments
Source: PLoS Comput Biol. 2022 Dec 27;18(12):e1010244. doi: 10.1371/journal.pcbi.1010244 (PMC9829172; doi:10.1371/journal.pcbi.1010244)
Supplement: Attachment — Submitted filename: plos_comp_bio_reviewer_responses.pdf [file pcbi.1010244.s004.pdf]

Dear Editor,

We are delighted to submit our revised manuscript “Fine-scale diversity of microbial communities due to satellite niches in boom and bust environments”.

In line with the insightful suggestions of both reviewers, we have heavily edited the manuscript, including new analytical calculations for coexistence probability, the timescale of environmental booms, and yield differences, among many others. We have also substantially improved the Introduction and Discussion to clarify the connection between our work and previous studies, including more than a dozen new citations and references. We have also corrected typographical and grammatical errors to improve the readability of the text, as suggested by both referees.

Below, we respond to all reviewer comments point by point (our responses in red).

We now believe that, with these changes, our manuscript is now ready for publication at *PLoS Computational Biology*. We hope you will agree with our assessment.

On behalf of all authors,
Akshit Goyal
Massachusetts Institute of Technology

Reviewer #1

In their manuscript “Fine-scale diversity of microbial communities due to satellite niches in boom-and-bust environments”, Wang et al. examine the role of temporal niches and R-K like tradeoff in sustaining fine-scale diversity using simple models. The manuscript is well written and is addressing an interesting question. The models are simple but illustrate the general point the authors wish to make. I think the manuscript makes a timely point that is of interest to the community studying microbial ecology and for this reason is potentially well-suited for publication in PLoS Comp Bio.

However, I do feel that the manuscript is currently quite minimal. It is a good starting point, but I would like to see a number of points addressed more systematically. Currently, the models essentially reproduce intuitions first derived using graphical arguments by Levins in the context of Monod kinetics. The derivations are solid and calculations are correct. I especially like the arguments in section SI 1.4. A strong point of the current approach is that one can understand the coexistence region mechanistically at least in these simple settings. However, there are a lot of specialized assumptions made.

We thank the reviewer for their encouragement and for suggestions and feedback, incorporating which has substantially improved our manuscript.

1. Please move S3 to main text. Otherwise, it is harder to follow. I would like to understand what happens when the growth rate (dN/dt) and the impact vector (dc/dt) are not exactly identical with opposite sign. We know that from the chemostat case that this assumption corresponds to the existence of a minimization principle. In general, we expect these to be correlated but not exact. Technically, the conservation law in S4 will be much more complicated and it would be interesting to at least understand when does the coexistence region get bigger or smaller.

We would like to thank the reviewer for this suggestion. In the revised manuscript, we have moved the resource dynamics equation to the main text (equation 2), and studied the case when growth rate and consumption are not identical with opposite sign, i.e., when resource-to-biomass yields are different from unity (Supplementary Text, section 1.3). Interestingly, we find that for the case of two

strains, having different yields does not affect the coexistence region, though this can change for > 2 strains.

2. Another interesting question is what happens if we no longer allow all the resources to be depleted and instead change time-scale of booms? How does co-existence region change? Technically, this seems to be assumed in derivation leading to S6.

This is an excellent suggestion. In the revised manuscript, we have studied the effect of a finite dilution period, T, on coexistence, and discussed the key results in the main text (equations 3 to 6). Equation 3 shows exactly how the coexistence region depends on the resource concentration at the end of each growth cycle, c_f (when T is arbitrarily large, c_f tends to 0), and equation 4 relates c_f to the time-scale T. It stands to reason that the coexistence region decreases as T decreases, eventually disappearing for small enough T.

3. In Equation S12, I am not sure what the small parameter is. I think it is 1/D. However, I cannot tell if other approximations are also being made (do I have to assume zeroeth order in K/c_o as well?). I would like to see corrections to next order in 1/D to better understand this. I am always a little confused what is held fixed and what is changing in the perturbations. This should not be too hard but may add some intuition to the problem.

Once again, we thank the reviewer for the insightful comment, which prompted us to revisit this calculation and more carefully discuss the approximations involved. In the revised manuscript, we have elevated these results to the main text, highlighting our assumptions, while the Supplementary Text contains the derivations. The small parameter is K/c_0, and we also assume that the time period between dilutions, T, is arbitrarily large, such that c_f (see previous reply) is 0. In the case when the difference between the two Ks, $(K_B - K_A) << K_{A, B}$, we obtain the simplified quadratic expression in equation (6), otherwise we obtain the lengthier expression in equation (S21).

4. Is there a way to derive a general scaling for the volume of the co-existence region as a function of number of species that need to co-exist. It seems like this might be possible from SI section 2. I tried and it was not super obvious to me but the authors clearly have thought about the problem much more than me.

There certainly is! We thank the reviewer for encouraging us to think about this deeply, which helped us realize that when the g and K parameters are uniformly distributed, we can exactly calculate the probability of n randomly chosen strains to coexist with each other. In the revised manuscript, we have included a new section titled “Simplified model and the statistics of coexistence”, where we highlight this result (the coexistence probability decreases faster than exponential, see equation (7)), and detailed its derivation in the Supplementary Text, section 3.2.

Minor Points.

1. In deriving S6, would be useful to say assume all resources are always depleted to derive.

Corrected. As discussed above, we have included an explicit section detailing what happens when resources are not fully depleted.

2. It would be helpful in derivation of S8-S10 to make connection to the idea of invasion fitness in static literature that the idea is to find when both species can invade each other at small numbers.

Thank you for this suggestion. We have now explicitly made the connection with invasion fitness, both in our derivation as well as briefly in the main text to appropriately cite and credit the work.

Reviewer #2

In this manuscript, the authors examined the extent that Monod growth in an environment with a single limiting resource permits coexistence. The authors use a mixture of simulation and analytic means to successfully demonstrate the existence of coexistence and compare their results to semi-quantitative results in the literature (e.g., the number of strains in the same host ~O(1)). I very much enjoyed reading the paper and think that it makes a worthwhile contribution to microbial ecology. However, there are many points that I think need to be addressed before this manuscript is accepted for publication. Given that there are no line numbers, I have split my comments into "major" and "minor" sections, and list comments for a given page within each section. If the editor decides that another round of revision is required, could the authors please provide a version of the manuscript with continuous line numbers?

We thank the reviewer for their extensive feedback and the time and effort they spent in offering us these valuable suggestions. Below, we respond to the criticisms point-by-point, and hope the reviewer now believes that the manuscript is ready for publication. The revised manuscript contains line numbers.

Major comments

General

- I may have missed it, but the authors frequently refer to "satellite niches" throughout the manuscript, but do not provide an explicit definition. It would help the reader if this term was defined when first used in the manuscript as well as in the abstract. It is also not clear to me what they mean by this term. Is it a short period of time within a given transfer cycle where a given strain has a higher growth rate due to the current resource composition?

We agree with the referee and apologize that satellite niches were not explicitly defined. We have rewritten multiple sections of the Introduction to emphasize the definition of satellite niches and the context in which they might appear.

Lines 44-55: *"Thus, the observation of fine-scale diversity suggests that every resource might contain multiple niches: a primary niche and several "satellite" niches, ready to be occupied by closely related strains, enabling their coexistence. Here, the primary niche is occupied by a strain most competitive at high nutrient concentrations, and each "satellite" niche is occupied by a strain which is more competitive in a specific range of lower nutrient concentrations. Satellite niches are not expected in stable environments, where resources are supplied con- tinuously at a constant rate and reach a fixed concentration, such as in a chemostat. In these conditions, competitive exclusion guarantees that only the strain best adapted to the steady-state concentration will be able to survive [13]. In contrast, in fluctuating environments where nutrient concentrations change in time, the existence of satellite niches remains a possibility [17, 18, 19]. Indeed, in the extreme case of environmental fluctuations, i.e., in boom-and-bust cycles, where re- source concentrations may vary over orders of magnitude, there is ample opportunity for multiple strains to coexist [20, 21]."*

- The authors seem to imply that coexistence in serial dilution experiments is contradictory (first sentence of the abstract). While I understand how the discrete nature of a serial dilution experiment can give rise to boom-and-bust dynamics within a given transfer cycle, its ability to do so depends on the details of the experiment. It is not clear to me whether this study examined a within-transfer dynamic that alters community dynamics across many transfers. I suppose that this could be phrased as "necessary, but not sufficient".

Throughout the manuscript, we only examine the dynamics of a single steady state growth cycle, reached after multiple transfers. Using simulations, we show that the system indeed reaches such a steady state, where each growth cycle is identical to the next one. We did not focus on how the

within-cycle dynamics change from initial conditions to the steady state. However, the serial dilution experiments that we compare our results with also reach a similar steady state, where multiple strains appear to coexist. In light of this, we feel that the comparison between the aforementioned experiments and our model is fair, and does not need to be phrased as “necessary, but not sufficient”.

- The research is solid, but the scholarship needs considerable work. There are only 19 references in this paper, 4 of which are authored by the authors. The authors cite a few essential texts, but the references are too sparse for a theoretical paper on a topic with as rich a background as resource consumption and a topic as new and exciting as microbial strain structure. By expanding their scholarship, the authors have an opportunity to strengthen the impact of this solid research effort. For example, they primarily examine how the maximum rate of growth and substrate affinity for a given pair of species constrain coexistence, a phenomenon which bears striking similarity to the “growth-efficiency trade-off” in the more physiology-focused areas of microbial ecology (Litchman et al., 2015; Muscarella et al., 2020; Roller et al., 2016). This is an instance where drawing a connection to other areas of the literature would increase the generality of the authors results. I have attempted to list other references throughout this review that may help the authors expand the scholarship of their work.

We thank the reviewer for these references, and apologize that the first version did not cite the literature sufficiently. In the revised manuscript, we have significantly expanded the references and citations to appropriately credit past work and make connections with them.

- I would recommend not using words like “obviously”, “easily seen”, etc. when describing mathematical details. It can unintentionally discourage readers,

We agree. We have corrected these terms wherever we saw them.

- Throughout the manuscript the authors state that their result, where more than three strains are unlikely to coexist, is in agreement with empirical data. I think that this result should come with the caveat that we are typically only able to detect strains in observational sequencing studies that have reached frequencies of ~O(10^-2). Given that the number of cells of a given species in the human gut can range from the millions to billions (Zhao et al., 2019), it is possible that there is substantial strain structure that we have not yet detected. This limitation has been pointed out in the literature (Shoemaker, 2022). Therefore, I think it is worth noting in this study that the authors provide a model that can explain strain coexistence **as we currently know it.**

We agree. We have added the suggested phrase or riders with similiar meaning (“to our knowledge”, e.g.) to the text where we found it relevant.

Pg. 1

“This is because the allowed parameter space for the coexistence shrinks significantly with the number of strains that coexist.”

- The authors mention parameter space in the Author Summary, on pg. 4 and illustrate if in Fig. 1c, but it is not discussed at length. Could the authors quantify the parameter space relative to the total space vs. the number of coexisting strains?

We thank the reviewer for this suggestion. In the revised manuscript, we have included new analytical results from a simplified version of our model quantifying exactly how the parameter space allowing coexistence shrinks faster than exponential with the number of strains (new section “Simplified model and the statistics of coexistence”).

Pg. 2

“Remarkably, this wide-ranging diversity can persist even in well-controlled laboratory settings, containing alternating cycles of exponential growth, followed by a transfer to fresh media after dilution by a large factor.”

- I do not think that the existence of diversity in serially-transferred communities constitutes the label “remarkable”. Typically, the inverse of the dilution rate is smaller than the total number of cells in a microbial ecology or evolution experiment by many orders of magnitude. And while I am unaware of a study that compiles dilution rates of these experiments, my understanding is that it is typically $O(10^{-3}$-$10^{-2})$. I

We have removed the word “remarkably” from the sentence.

Pg. 4

“both strains could coexist, one can measure area of the space of parameters c0 and D where both strains survive (the region between the black lines in Fig. 1c)”

- It would help the reader if the authors could provide some estimates of the area of parameter space where coexistence is possible.

The region of coexistence is unbounded, so the area of the region is ill-defined. In the revised manuscript, we use the simplified model to quantify the probability of coexistence of *n* strains, and show how it decreases with *n*. We hope this quantification and the associated calculation in the Supplementary Text (section 3.2) helps the reader get an idea of the likelihood of coexistence.

“In the rest of this manuscript, we will refer to this coexistence region for strain A as its “shadow””

- I don’t understand why the term “shadow” is needed. As a reader, the term obfuscates the message, which is not the authors’ intention. I would recommend referring to this coexistence region as “environment dependent coexistence space” throughout the manuscript or some other term that captures the fact that this coexistence region depends on the choice of c0 and D, if I’m correctly understanding the manuscript.

We understand the reviewer’s concern regarding the unorthodox terminology. Our intention with the term “shadow” was to provide a visually evocative shorthand for the pairwise coexistence region associated with any strain. Of course, “environment dependent coexistence space” is correct, but we feel more comfortable using “shadow” and appropriately defining it to avoid being overly wordy in the text.

“However, simulations of our model with three strains revealed that this is not the case (Fig. S1); instead the region where all three strains truly coexist with each other (shown in dark grey, Fig. 2b) is a smaller sub-region of the intersection of both pairwise shadows (Fig. S1).

In hindsight, this is not surprising. Indeed, two strains lying in the shadow of each other only ensures their pairwise coexistence. Generally, pairwise coexistence between all pairs does not guarantee that all three strains will be able to simultaneously coexist..”

- Please elaborate on why pairwise coexistence between all pairs does not guarantee coexistence between three strains for the reader.

In the revised manuscript, we have now explained using an example from competitive exclusion why pairwise coexistence between all pairs does not guarantee coexistence between three strains.

Lines 138-141: *“As an illustrative example, in a chemostat model with two resources, competitive exclusion may allow strains to exist in pairs, but prohibit them from all coexisting together (since in this case, the number of strains cannot exceed the number of resources).”*

Pg. 8

"In this paper, we showed that in time-varying environments, the competitive exclusion principle can be broken through the formation of a few satellite niches alongside the primary resource niche"

- The paper would be strengthened by the authors discussing the competitive exclusion principle in greater depth, its relevance in microbial ecology, and how their study differs from prior studies in the Introduction.

We have discussed how our study differs from prior studies in the Discussion, about how our work adds to cases where time-varying environments allow competitive exclusion to be violated.

Lines 237-243: " *Specifically, our work provides new mathematical expressions for the coexistence criteria applicable to two strains, connecting several relevant parameters, especially the time period between successive growth-dilution cycles, the dilution factor, as well as the growth parameters of the strains (equation (3–5)). Another new aspect of our study relative to prior work in this area, in particular Ref. [21], is the coexistence of multiple (≥ 3) strains in environments with more than one supplied resource (primary niches), such that species could also specialize to change the ratio in which they consume different resources, i.e., change how they allocate their enzyme budgets."*

"In these domesticated communities, between 1 and 2 closely related strains of the same species were found to coexist over multiple (~ 70) boom-and-bust (serial dilution) cycles, consistent with the predictions of our model."

- An advantage of serial dilution is that under certain experimentally imposed conditions (e.g., large number of transfers, population remains in near-exponential growth between transfer), you can coarse-grain over the discrete nature of the experiment and view the dynamics as approximately continuous in time. In Ref. 11 the senior author used a serial dilution ratio of 1:2, which gives the experiment a timescale of log2(2) [gens/day] *70 [gens] = 70 generations (if exponential growth is an appropriate assumption). So, this seems like an experimental scenario where boom-and-bust details of a given cycle might not matter because few generations occur over the course of a given transfer cycle. My question then is when can you coarse-grain over the within-cycle dynamics that the authors examined, if at all? This may be too big a question to answer in a revision, but I think a brief discussion would help the paper appeal to a wider audience.

In the revised manuscript, we have now included Supplementary Text (section 4) explaining the conditions under which within-cycle dynamics can be coarse-grained and replaced with a chemostat.

"Following in the footsteps of Good et al. [9], we adapted our model to include the possibility of small variations in resource budget allocation by closely related strains."

- I am unable to follow this sentence. My understanding is that the authors chose parameter combinations and identified the parameter space where coexistence was permitted, through computational and analytic means. While the maximum rate of growth can be negatively related to the substrate affinity, and the authors focused on parameter combinations where a strain is either an r or K-specialist (as opposed to both, a Darwinian monster), there was no explicit resource budget in the model that could constrain the relationship between these two parameters.

We understand the confusion, and wish to clarify that the statement in question was about an extension of our model which did indeed include an explicit resource budget (see Fig. S2). In this version, there are two resources (as opposed to one in the vanilla model), and each strain derives some fraction *x* of its growth from one resource, and (1 - *x*) from the other. By varying x, we generate species which budget resources differently, and each strain within a species can further have different maximal growth rate (g_{max}) and affinity (K) on each resource. In the revised manuscript, we

have clarified to readers the fact that we are talking about an extension of the model with a resource budget (lines 241-243).

Lines 241-243; "*Another new aspect of our study relative to prior work in this area, in particular Ref. [21], is the coexistence of multiple (≥ 3) strains in environments with more than one supplied resource (primary niches), such that species could also specialize to change the ratio in which they consume different resources, i.e., change how they allocate their enzyme budgets.*"

Pg. 9

"Their maximum growth rates were sampled independently from a normal distribution of N (1.0, 0.032) in hr−1 . Their substrate affinities were sample independently from a log-normal distribution of logNormal(log 4, 0.5 2) in a.u."

- Why were these choices made? I understand that substrate affinities introduce a logarithmic dependence between resource concentration and the rate of growth, but why should we expect substrate affinities to follow a lognormal distribution? And why are the standard deviation values so low? It is understandable that the exact distribution may not be known, but is there any empirical data that the authors could use to justify their choices of mean and variance?

We understand the reviewer's concern about our choice of parameter values. To our knowledge, we do not have data to help constrain the mean and variance of the distribution of parameters. In our model, the mean and variance were chosen arbitrarily for the sake of simulations. In the revised manuscript, we have clarified that the choices were arbitrary.

"https://github.com/maslov-group/Coexistence_of_g_and_K."

- The embedded link does not lead to the repo and the URL, as it is written, contains backshlashes that are not in the actual URL.

Thanks for pointing that out: these errors crept in due to LaTeX. We have corrected them.

Pg. 11

"This can happen because the Monod's dynamics, under which the logarithmic growth rate for a species B_{i} is given by g_{i} c/(c+ K_{i})"

- The term "logarithmic growth rate" may be unclear for the typical reader. If I am understanding the manuscript correctly, the term is referring to the logarithmic curve that growth rate takes on as resource concentration increases by multiple orders of magnitude. A new reader might think that you are referring to the logarithm **of** the growth rate. I don't think this is the author's intention, but it would be straightforward to refer to g_{i} c/(c+ K_{i}) as simply "the growth rate". The term "logarithmic growth rate" is also used on pgs. 17 and 18.

Following the referee's suggestion we have replaced the term "logarithmic growth rate" with "growth rate" throughout the manuscript.

"No degeneration in terms of g, K–parameters is required for the coexistence to be achieved: even if narrow, it is still not a null-dimensional curve in the g, K-plane, but rather a region of parameters."

- What do the authors mean by "degeneration" and "null-dimensional curve"? Would "null-dimensional curve" be a case where the dependence on environmental parameters falls out from the derivation?

We added an explanation: by degeneration we meant the requirement that the parameters defining growth coexisting strains or species satisfied a specific functional dependence g = g (K). Were it the

case, those parameters plotted in the g, K-plane would have fallen on a null-dimensional curve rather than spanning a region with finite area. (Hence two randomly chosen microbes would have a zero chance to coexist.) In the revised manuscript, we have explicitly defined “degeneration” as a specific functional dependence and removed the term null-dimensional curve since it was unnecessary.

- The use of the term N_{10} is confusing, as is c_{0}, since the authors use numerical subscripts to refer to strains. Consider using parentheses to denote initial conditions (i.e., $c(0)$).

We thank the referee for pointing out a possible source of confusion. We have replaced the notations like N_{10} with $N_{1, 0}$ etc, so that they could not be confused with the identification numbers of strains or species. As for c_0 denoting the initial resource abundance at the beginning of a dilution cycle, we have checked that it is used only where there is a single resource present, to avoid confusion.

Pg. 14

“If there is a biological reason for a species to be able to lessen considerably its K– value at the expence of decreasing its own growth rate by a tiny little bit, one can observe two strains of the same species surviving on a single source in an environment with boom and bust cycles”

- This seems like a missed opportunity to talk about trade-offs between growth rate and efficiency. It may be outside the scope of this paper, but I think describing this trade-off and citing the relevant literature would strengthen the transition from species to strain-level coexistence (e.g., Roller et al., 2016). One potentially interesting example is that the trade-off between growth and efficiency seems stronger at **lower** taxonomic orders (Muscarella et al., 2020), which could extend to the strain-level.

We thank the reviewer for this suggestion and for the references. In the revised manuscript, we have added a paragraph to the Discussion where we talk about trade-offs and cite the appropriate references as suggested.

Lines 266-275: “*Our work intriguingly suggests that a trade-off between Monod growth parameters (gmax and 1/K) might promote coexistence, and might be observed among coexisting microbial strains in natural communities. Past work [34, 35] has indicated similar trade-offs between maximal growth rate and efficiency, or yield (the fraction of environmental carbon converted to biomass), some even suggesting that these trade-offs become more pronounced at lower taxonomic levels [34]. While the yield or efficiency parameter in these studies does not influence two-strain coexistence in our model (Supplementary Text), a trade-off between maximal growth rate (gmax) and affinity (1/K) in our model has been discussed and reviewed in Ref. [36], suggesting that they might be present in natural bacterial and phytoplankton communities and promote coexistence. Future work examining such a trade-off and its causal implication for coexistence would be fruitful.*”

Pg. 14.

“Thus, in this limit, the coexistence range for the value of the ratio g1/g2 is thin, its width r given by”

- Could the authors provide an expression for the width of the coexistence range relative to the total width for Eqs. S14 and S15? I think that would help the reader get a handle on the narrowness of the range.

As S14 and S15 were related to a special case of S11, we included a commentary immediately below S11: “*Note that, as $g_2 < g_1$, the width of the range of the value $0 < g_2 / g_1 < 1$ favoring coexistence should be evaluated with respect to 1.*”

Pg. 17.

“Comparing this with γ is easily reduced to comparing 0 with $(\gamma x-1)(1-\gamma) > 0$”

- Could the authors elaborate on why this is the case?

Upon the referee’s suggestion we have elaborated on this in the manuscript.

Pg. 18.

“Real Monod’s dynamics does not work this way, and yet there are similarities, especially when the K–constants of both competitors are very different.”

- Please briefly explain how Monod dynamics differ for the reader.

We now explain the difference.

Minor comments

General

- The authors periodically use “food” and “resource” in the same paragraph, seemingly interchangeably. It is unclear if there is a reason for this. If not, please stick with “resource”.

We have changed all references to use “resource”.

- The authors use both hyphenated and non-hyphenated forms of “boom-and-bust” throughout the manuscript. Please stick with one.

Corrected.

- Throughout the manuscript the authors use “r.h.s.” with and without spaces.

Corrected.

Pg. 1

“we propose that by differentiating on Monod parameters characterizing microbial growth rates in high and low nutrient conditions, strains can coexist in patterns similar to those observed.”

- Should it be “differentiating Monod parameters that characterize microbial growth rates…..”?

We have rewritten this to “differentiating along Monod parameters”.

“Recent genomic data has exposed to us the remarkable spectrum ”

- The word “exposed” is a strange choice for this sentence, consider “….has revealed the remarkable….”.

Reworded.

“provided that the environments vary in time…”

- While generality is important, I think here it would clarify the Author Summary if the authors named the feature of the environment that is pertinent to this study.

Added “specifically, resource concentrations” to specify the feature of the environment.

“These scales range from separate kingdoms (e.g., archaea and bacteria) all the way down to closely-related strains of the same species [8]”

- If the authors want to emphasize the difference in scales, they could use Domain in instead of Kingdom.

Corrected.

Pg. 2

“Indeed, in the extreme case of environmental fluctuations, i.e., in boom-and-bust cycles, the selection criteria for strains occur along two separate dimensions: one for strains that grow well at high nutrient concentrations (the “boom” phase), but poorly at low nutrient concentrations, and the other for strains that dominate in the “bust” phase, at much lower nutrient concentrations”

- Could the authors identify and cite some appropriate empirical examples to back up this generalization?

We meant for this to be a statement substantiated by a model, such as ours. We have rephrased the sentence and added citations.

Lines 53-55: “*Indeed, in the extreme case of environmental fluctuations, i.e., in boom and bust cycles, where resource concentrations may vary over orders of magnitude, there is ample opportunity for multiple strains to coexist [20, 21].”*

“The coexistence of distantly related community members, such as different species or kingdoms, can be readily explained via niche theory, which suggests that each species can occupy a different niche, e.g., by specializing on a different resource, allowing everyone to coexist.”

- I think that it is necessary to cite some of the vast literature on niche theory in this sentence, even if it’s just Chase and Leibold’s book “Ecological Niches”. There is also a more recent paper focused on applying niche theory to microbes (Baquero et al., 2021). While niche theory can be an overwhelming area of theoretical ecology, I think identifying and citing the relevant literature here would strengthen the paper.

Corrected. We thank the reviewer for these references, and apologize again for the lack of references in our manuscript.

“selection criteria for strains occur along two separate dimensions”

- While ecology and evolution are co-occurrent at the strain level, in this case it seems like the authors are referring to “ecological selection” (as in Vellend (2010)). If so, consider specifying this for the reader.

The rewritten sentence no longer talks about selection.

Lines 53-55: “*Indeed, in the extreme case of environmental fluctuations, i.e., in boom and bust cycles, where resource concentrations may vary over orders of magnitude, there is ample opportunity for multiple strains to coexist [20, 21].”*

Pg. 3

- Consider including logarithmically spaced axis ticks for Fig. 1c. Both 1b and 1d have these ticks, and it makes their lack stand out for Fig. 1c.

Corrected.

- The authors used a linear heatmap to plot relative abundance in Fig. 1c. Since a number of macroecological patterns are examined using the logarithm of relative abundance, I wonder what this plot looks like with a logarithmic heatmap. Finally, the use of a diverging colormap is a strange choice. While coexistence requires strain B to have a relative abundance between zero and one, it is not clear why the authors chose to have the colormap diverge at 0.5. Did they run simulations for parameter regimes where their theory predicts that coexistence should occur at 0.5? If not, consider using a sequential colormap. Is so, it should be stated in the figure caption.

Thanks for this suggestion. We believe the logarithmic axis would not significantly change the heatmap, since it would better highlight the region where the abundance of strain B is very small (right above the bottom black border marked "coexistence"), which happens to be a very narrow region in the plot. The rest of the heatmap would largely remain the same, though the reviewer is right to point out that there is nothing special about a relative abundance of 0.5.

Pg. 4

"both strains could coexist, one can measure area of the space of parameters"

- Should it be "the area of the space"?

Corrected.

"Note that the width of this region depends only mildly (logarithmically) on the dilution factor D compared with its strong, inverse dependence on the resource concentration c0, suggesting that coexistence of strains should be possible for a broad range of environmental fluctuations"

- I would just say "logarithmically" here for *D* and replace "strong" with linear for c0.

Corrected. Our new calculation shows that it is roughly logarithmic, and truly so for large *D*.

Pg. 5

- Sub-figure label "c" is right in the middle of the figure.

Corrected.

Pg. 7

- Is there a reason why percentages are plotted on the barchart in Fig. 3e? Instead, I would recommend plotting 95% CIs of their simulation results.

In Fig. 3e, we show results from one simulation, whose dynamics are deterministic and steady state abundances reach a fixed value. Thus we feel that confidence intervals are not needed for this panel. The percentages are plotted on a barchart to show that the relative abundances of the three coexisting strains are roughly of the same order of magnitude, not heavily skewed towards one or two strains.

Pg. 8

"investigating how nonlinearities in resource-dependent growth can lead to violations of the competitive exclusion principle"

- Specify where the nonlinearity comes from. My understanding is that they are referring to the form of the Monod growth equation, this should be made explicit.

Specified.

Pg. 9

“we present every strain at equal amount of 1 unit at the beginning.”

- This description is unclear. Please explain what 1 unit is.

Explained.

“Their maximum growth rates are 0.55 hr−1 and 1.0 hr−1 respectively, with substrate affinity being 0.01 a.u. and 4.0 a.u. In Fig.2b, we introduced an invader strain C whose maximum growth rates vary between [0.6, 0.7] hr−1 and substrate affinity vary between [0.25, 1.05] a.u.”

- Please briefly elaborate on the rationale behind these chosen values.

Explained.

Pg. 11

“While in the case of the constant nutrient supply”

- Should it be “While in the case of constant nutrient supply”?

Corrected.

“can never exceed the number of (generalized) sources”

- Is “sources” supposed to be “resources”? And is “generalized” refer to “substitutable” in that two strains/species **can** consume the resource, though with different Monod parameters? If so, I would clarify that point.

We have replaced “sources” with “resources” and clarified our usage of the term “generalized”.

- Please define c^{*} for the reader.

We have added the reference to the equation in which c^{*} is defined.

“under some conditions may make it D–fold simultaneously with B_{1}”

- Is this supposed to be “…may make it grow D–fold simultaneously….”?

We have rewritten this particular sentence.

“single source”

- Replace with “single resource”.

Done.

Pg. 12

- The caption for Fig. S1 needs additional detail.

We have now provided additional detail for the Fig. S1 caption: “*Heatmaps showing the final community diversity (number of strains) as a function of the growth parameters g_C and K_C. Given a strain C with variable g_{max} and K along with fixed strains A and B, the heatmap shows the number of coexisting species in the steady state of serial dilutions. Here, the region above*

the blue line represents where C drives A out of the steady state, and the region above the red line represents where B is driven out by C. The green regions represent areas where more than one strain can coexist. (b) shows a zoomed-in view of the boxed region in (a), highlighting the region where two (light green) and three (dark green) strains can coexist."

- Is this the same variation in resource partitioning that was used for Fig. S2b? If so or if not, please provide this detail in the legend. Also, please briefly describe the change in the Monod parameters in the legend.

Explained.

Pg. 13

"For the same reason the steady state initial abundance of the microbe for each dilution cycle should be…"

- This took me a few minutes to work out and I imagine it may also happen to a typical reader. Please provide additional detail on how you arrived at the solution for N_{10}.

Done.

"we can use (??) to give dc/dt as we …"

- Missing equation reference.

We are grateful to the referee for pointing that out for us.

Pg. 14.

"…own growth rate by a tiny little bit…."

- What range of values is this?

We have clarified this.

", whenever the nutrient supply come somewhere"

- Replace "come" with "is".

Done.

Pg. 15.

"would have to concern oneself with the sign of the expression in the brackets, which we rewrite as"

- The expression is in parentheses, not brackets.

Corrected.

Pg. 16.

"That is what makes ∂f(x, y, D)/partialx from"

- "partialx" did not compile correctly.

We have corrected this.

“Rewrite (S11) in terms of x, y, γ. As, again..”

- It would be helpful for the reader if the authors described the motivation for this derivation.

We have done that.

Pg. 17.

- Please write out “l.h.s.” in Eq. S30.

Done.

“so that γ can be indeed coaxed into the interval between...“

- Do the authors mean to say that once can always choose some value of γ in this interval?

We thank the referee for pointing out an ambiguity in our explanations and have added a passage that should eliminate the confusion:

“ (Let us emphasize again that $\gamma = g_2 / g_1$ is a fixed value in this subsection. We chose two strains or species B_1 and B_2 and look for the environmental parameters c_0, D that would ensure that the l. h. s. of (\ref{criterion dimensionless}) is less than γ while at the same time r. h. s. of (\ref{criterion dimensionless}) is greater than γ. While we cannot offer an analytical solution providing the pairs (c_0, D) thus favouring coexistence of B_1 and B_2, we can prove that such pairs do exist and offer a way to find them numerically.)”

“that would be still a dynamics that is characterised by two parameters”

- I’d recommend replacing with “there would still be dynamics that is characterised by…”.

Done.

Pg. 20.

- For reference, it would be helpful for there to be a horizontal dashed grey line at 0.5 on the y-axis to remind the new reader about the meaning behind K_{A}^{Monod}.

Added.

- Fig. S4 is slightly cut-off on the y-axis.

Corrected.

Pg. 21

- “each providing its own value of the (exponential) growth rate for each microbe” It is unclear why “exponential” is in parentheses. Are the authors implying that their analysis is applicable to forms of growth rate functions beyond what they examined in this paper?

Indeed. In the simplified model, we assume that in each region of concentration where growth occurs, it occurs exponentially with a fixed growth rate. Any monotonic growth rate function may be broken up stepwise in such a way, and our analysis would be applicable to all such curves. In the revised manuscript, we clarify this generalizability of the simplified model to the reader.

Lines 627-628: “*Indeed, any monotonically increasing growth rate function may be approximated this way, by an arbitary number of resources and steps.*”

Corrected.

- "the number of survivors matching the number of sources" replace "matching" with "match".

Done.

- "have different source preferences" should "source" be "resource"?

Replaced.

- "one of them starts consuming the resource number" remove "the".

Removed.

- "In our case, the species are bound to have the same food preferences, and in this case a multiple coexistence is much harder (though possible) to arrange." Are the authors able to get some rough estimates on the degree of similarity of resource preferences? Is this basically a partition problem, where the [sic]

One can say that it is a partition problem indeed. Strictly speaking, when performing the partition, one should use the axis c(t) instead of t, measuring time in the units of resource consumed. What authors mean to say is that, partitioning time or rather resource abundance as dependent on time into small intervals, one can assume that a strain or species grows purely exponentially with the same growth rate g c(t) / (c(t) + K) through each interval and than undertaking a diauxic switch to a resource on which it grows with a slightly less growth rate. When there are real multiple resources and true diauxie is involved, species B_1 may consume resource 1 never affecting the abundance of resource 2, while species B_2 may even grow solely on resource 2 through the whole dilution period, never interfering with the abundances of other resources. We cannot have that building our analogy. All the species that are present in the environment deplete the (virtual) multiple resources one by one, switching from one to another simultaneously and in the same order. Thus, in our case, the degree of similarity is absolute.

- Shorten "the species" to "species" and remove "a" from the sentence.

Done.

References

Baquero, F., Coque, T. M., Galán, J. C., & Martinez, J. L. (2021). The Origin of Niches and Species in the Bacterial World. *Frontiers in Microbiology*, *12*. https://www.frontiersin.org/article/10.3389/fmicb.2021.657986

Litchman, E., Edwards, K. F., & Klausmeier, C. A. (2015). Microbial resource utilization traits and trade-offs: Implications for community structure, functioning, and biogeochemical impacts at present and in the future. *Frontiers in Microbiology*, *6*, 254. https://doi.org/10.3389/fmicb.2015.00254

Muscarella, M. E., Howey, X. M., & Lennon, J. T. (2020). Trait-based approach to bacterial growth efficiency. *Environmental Microbiology*, *22*(8), 3494–3504. https://doi.org/10.1111/1462-2920.15120

Roller, B. R. K., Stoddard, S. F., & Schmidt, T. M. (2016). Exploiting rRNA operon copy number to investigate bacterial reproductive strategies. *Nature Microbiology*, *1*(11), 1–7. https://doi.org/10.1038/nmicrobiol.2016.160

Shoemaker, W. R. (2022). *A macroecological perspective on genetic diversity in the human gut microbiome* (p. 2022.04.07.487434). bioRxiv. https://doi.org/10.1101/2022.04.07.487434

Vellend, M. (2010). Conceptual Synthesis in Community Ecology. *The Quarterly Review of Biology*, *85*(2), 183–206. https://doi.org/10.1086/652373

Zhao, S., Lieberman, T. D., Poyet, M., Kauffman, K. M., Gibbons, S. M., Groussin, M., Xavier, R. J., & Alm, E. J. (2019). Adaptive Evolution within Gut Microbiomes of Healthy People. *Cell Host & Microbe*, *25*(5), 656-667.e8. https://doi.org/10.1016/j.chom.2019.03.007
