# Supplementary material for: Fine-scale diversity of microbial communities due to satellite niches in boom and bust environments
Source: PLoS Comput Biol. 2022 Dec 27;18(12):e1010244. doi: 10.1371/journal.pcbi.1010244 (PMC9829172; doi:10.1371/journal.pcbi.1010244)
Supplement: Attachment — Submitted filename: 20220604_plos_comp_bio.docx [file pcbi.1010244.s002.docx]

In this manuscript, the authors examined the extent that Monod growth in an environment with a single limiting resource permits coexistence. The authors use a mixture of simulation and analytic means to successfully demonstrate the existence of coexistence and compare their results to semi-quantitative results in the literature (e.g., the number of strains in the same host ~O(1)). I very much enjoyed reading the paper and think that it makes a worthwhile contribution to microbial ecology. However, there are many points that I think need to be addressed before this manuscript is accepted for publication. Given that there are no line numbers, I have split my comments into “major” and “minor” sections, and list comments for a given page within each section. If the editor decides that another round of revision is required, could the authors please provide a version of the manuscript with continuous line numbers?

**Major comments**

**General**

- I may have missed it, but the authors frequently refer to “satellite niches” throughout the manuscript, but do not provide an explicit definition. It would help the reader if this term was defined when first used in the manuscript as well as in the abstract. It is also not clear to me what they mean by this term. Is it a short period of time within a given transfer cycle where a given strain has a higher growth rate due to the current resource composition?
- The authors seem to imply that coexistence in serial dilution experiments is contradictory (first sentence of the abstract). While I understand how the discrete nature of a serial dilution experiment can give rise to boom-and-bust dynamics within a given transfer cycle, its ability to do so depends on the details of the experiment. It is not clear to me whether this study examined a within-transfer dynamic that alters community dynamics across many transfers. I suppose that this could be phrased as “necessary, but not sufficient”.
- The research is solid, but the scholarship needs considerable work. There are only 19 references in this paper, 4 of which are authored by the authors. The authors cite a few essential texts, but the references are too sparse for a theoretical paper on a topic with as rich a background as resource consumption and a topic as new and exciting as microbial strain structure. By expanding their scholarship, the authors have an opportunity to strengthen the impact of this solid research effort. For example, they primarily examine how the maximum rate of growth and substrate affinity for a given pair of species constrain coexistence, a phenomenon which bears striking similarity to the “growth-efficiency trade-off” in the more physiology-focused areas of microbial ecology (Litchman et al., 2015; Muscarella et al., 2020; Roller et al., 2016). This is an instance where drawing a connection to other areas of the literature would increase the generality of the authors results. I have attempted to list other references throughout this review that may help the authors expand the scholarship of their work.
- I would recommend not using words like “obviously”, “easily seen”, etc. when describing mathematical details. It can unintentionally discourage readers,
- Throughout the manuscript the authors state that their result, where more than three strains are unlikely to coexist, is in agreement with empirical data. I think that this result should come with the caveat that we are typically only able to detect strains in observational sequencing studies that have reached frequencies of ~O(10^-2). Given that the number of cells of a given species in the human gut can range from the millions to billions (Zhao et al., 2019), it is possible that there is substantial strain structure that we have not yet detected. This limitation has been pointed out in the literature (Shoemaker, 2022). Therefore, I think it is worth noting in this study that the authors provide a model that can explain strain coexistence **as** **we currently know it.**

**Pg. 1**

**“**This is because the allowed parameter space for the coexistence shrinks significantly with the number of strains that coexist.”

- The authors mention parameter space in the Author Summary, on pg. 4 and illustrate if in Fig. 1c, but it is not discussed at length. Could the authors quantify the parameter space relative to the total space vs. the number of coexisting strains?

**Pg. 2**

“Remarkably, this wide-ranging diversity can persist even in well-controlled laboratory settings, containing alternating cycles of exponential growth, followed by a transfer to fresh media after dilution by a large factor.”

- I do not think that the existence of diversity in serially-transferred communities constitutes the label “remarkable”. Typically, the inverse of the dilution rate is smaller than the total number of cells in a microbial ecology or evolution experiment by many orders of magnitude. And while I am unaware of a study that compiles dilution rates of these experiments, my understanding is that it is typically O(10^-3^-10-^2^). I

**Pg. 4**

“both strains could coexist, one can measure area of the space of parameters c0 and D where both strains survive (the region between the black lines in Fig. 1c)”

- It would help the reader if the authors could provide some estimates of the area of parameter space where coexistence is possible.

“In the rest of this manuscript, we will refer to this coexistence region for strain A as its “shadow””

- I don’t understand why the term “shadow” is needed. As a reader, the term obfuscates the message, which is not the authors’ intention. I would recommend referring to this coexistence region as “environment dependent coexistence space” throughout the manuscript or some other term that captures the fact that this coexistence region depends on the choice of c0 and D, if I’m correctly understanding the manuscript.

“However, simulations of our model with three strains revealed that this is not the case (Fig. S1); instead the region where all three strains truly coexist with each other (shown in dark grey, Fig. 2b) is a smaller sub-region of the intersection of both pairwise shadows (Fig. S1).

In hindsight, this is not surprising. Indeed, two strains lying in the shadow of each other only ensures their pairwise coexistence. Generally, pairwise coexistence between all pairs does not guarantee that all three strains will be able to simultaneously coexist..”

- Please elaborate on why pairwise coexistence between all pairs does not guarantee coexistence between three strains for the reader.

**Pg. 8**

“In this paper, we showed that in time-varying environments, the competitive exclusion principle can be broken through the formation of a few satellite niches alongside the primary resource niche”

- The paper would be strengthened by the authors discussing the competitive exclusion principle in greater depth, its relevance in microbial ecology, and how their study differs from prior studies in the Introduction.

“In these domesticated communities, between 1 and 2 closely related strains of the same species were found to coexist over multiple (∼ 70) boom-and-bust (serial dilution) cycles, consistent with the predictions of our model.”

- An advantage of serial dilution is that under certain experimentally imposed conditions (e.g., large number of transfers, population remains in near-exponential growth between transfer), you can coarse-grain over the discrete nature of the experiment and view the dynamics as approximately continuous in time. In Ref. 11 the senior author used a serial dilution ratio of 1:2, which gives the experiment a timescale of log2(2) [gens/day] *70 [gens] = 70 generations (if exponential growth is an appropriate assumption). So, this seems like an experimental scenario where boom-and-bust details of a given cycle might not matter because few generations occur over the course of a given transfer cycle. My question then is when can you coarse-grain over the within-cycle dynamics that the authors examined, if at all? This may be too big a question to answer in a revision, but I think a brief discussion would help the paper appeal to a wider audience.

“Following in the footsteps of Good et al. [9], we adapted our model to include the possibility of small variations in resource budget allocation by closely related strains.”

- I am unable to follow this sentence. My understanding is that the authors chose parameter combinations and identified the parameter space where coexistence was permitted, through computational and analytic means. While the maximum rate of growth can be negatively related to the substrate affinity, and the authors focused on parameter combinations where a strain is either an r or K-specialist (as opposed to both, a Darwinian monster), there was no explicit resource budget in the model that could constrain the relationship between these two parameters.

**Pg. 9**

“Their maximum growth rates were sampled independently from a normal distribution of N (1.0, 0.032 ) in hr−1 . Their substrate affinities were sample independently from a log-normal distribution of logNormal(log 4, 0.5 2 ) in a.u.”

- Why were these choices made? I understand that substrate affinities introduce a logarithmic dependence between resource concentration and the rate of growth, but why should we expect substrate affinities to follow a lognormal distribution? And why are the standard deviation values so low? It is understandable that the exact distribution may not be known, but is there any empirical data that the authors could use to justify their choices of mean and variance?

“https://github.com/maslov-group/Coexistence\_of\_g\_and\_K.”

- The embedded link does not lead to the repo and the URL, as it is written, contains backshlashes that are not in the actual URL.

**Pg. 11**

“This can happen because the Monod’s dynamics, under which the logarithmic growth rate for a species B_{i} is given by g_{i} c/(c+ K_{i})”

- The term “logarithmic growth rate” may be unclear for the typical reader. If I am understanding the manuscript correctly, the term is referring to the logarithmic curve that growth rate takes on as resource concentration increases by multiple orders of magnitude. A new reader might think that you are referring to the logarithm **of** the growth rate. I don’t think this is the author’s intention, but it would be straightforward to refer to g_{i} c/(c+ K_{i}) as simply “the growth rate”. The term “logarithmic growth rate” is also used on pgs. 17 and 18.

“No degeneration in terms of g, K−parameters is required for the coexistence to be achieved: even if narrow, it is still not a null-dimensional curve in the g, K-plane, but rather a region of parameters.”

- What do the authors mean by “degeneration” and “null-dimensional curve”? Would “null-dimensional curve” be a case where the dependence on environmental parameters falls out from the derivation?
- The use of the term N_{10} is confusing, as is c_{0}, since the authors use numerical subscripts to refer to strains. Consider using parentheses to denote initial conditions (i.e., c(0)).

**Pg. 14**

“If there is a biological reason for a species to be able to lessen considerably its K− value at the expence of decreasing its own growth rate by a tiny little bit, one can observe two strains of the same species surviving on a single source in an environment with boom and bust cycles”

- This seems like a missed opportunity to talk about trade-offs between growth rate and efficiency. It may be outside the scope of this paper, but I think describing this trade-off and citing the relevant literature would strengthen the transition from species to strain-level coexistence (e.g., Roller et al., 2016). One potentially interesting example is that the trade-off between growth and efficiency seems stronger at **lower** taxonomic orders (Muscarella et al., 2020), which could extend to the strain-level.

**Pg. 14.**

“Thus, in this limit, the coexistence range for the value of the ratio g1/g2 is thin, its width r given by”

- Could the authors provide an expression for the width of the coexistence range relative to the total width for Eqs. S14 and S15? I think that would help the reader get a handle on the narrowness of the range.

**Pg. 17.**

“Comparing this with γ is easily reduced to comparing 0 with (γ x−1)(1−γ) > 0”

- Could the authors elaborate on why this is the case?

**Pg. 18.**

“Real Monod’s dynamics does not work this way, and yet there are similarities, especially when the K−constants of both competitors are very different.”

- Please briefly explain how Monod dynamics differ for the reader.

**Minor comments**

**General**

- The authors periodically use “food” and “resource” in the same paragraph, seemingly interchangeably. It is unclear if there is a reason for this. If not, please stick with “resource”.
- The authors use both hyphenated and non-hyphenated forms of “boom-and-bust” throughout the manuscript. Please stick with one.
- Throughout the manuscript the authors use “r.h.s.” with and without spaces.

**Pg. 1**

**“**we propose that by differentiating on Monod parameters characterizing microbial growth rates in high and low nutrient conditions, strains can coexist in patterns similar to those observed.”

- Should it be “differentiating Monod parameters that characterize microbial growth rates…..”?

**“**Recent genomic data has exposed to us the remarkable spectrum ”

- The word “exposed” is a strange choice for this sentence, consider “….has revealed the remarkable….”.

“provided that the environments vary in time…”

- While generality is important, I think here it would clarify the Author Summary if the authors named the feature of the environment that is pertinent to this study.

**“**These scales range from separate kingdoms (e.g., archaea and bacteria) all the way down to closely-related strains of the same species [8]”

- If the authors want to emphasize the difference in scales, they could use Domain in instead of Kingdom.

**Pg. 2**

“Indeed, in the extreme case of environmental fluctuations, i.e., in boom-and-bust cycles, the selection criteria for strains occur along two separate dimensions: one for strains that grow well at high nutrient concentrations (the “boom” phase), but poorly at low nutrient concentrations, and the other for strains that dominate in the “bust” phase, at much lower nutrient concentrations”

- Could the authors identify and cite some appropriate empirical examples to back up this generalization?

“The coexistence of distantly related community members, such as different species or kingdoms, can be readily explained via niche theory, which suggests that each species can occupy a different niche, e.g., by specializing on a different resource, allowing everyone to coexist.”

- I think that it is necessary to cite some of the vast literature on niche theory in this sentence, even if it’s just Chase and Leibold’s book “Ecological Niches”. There is also a more recent paper focused on applying niche theory to microbes (Baquero et al., 2021). While niche theory can be an overwhelming area of theoretical ecology, I think identifying and citing the relevant literature here would strengthen the paper.

“selection criteria for strains occur along two separate dimensions”

- While ecology and evolution are co-occurrent at the strain level, in this case it seems like the authors are referring to “ecological selection” (as in Vellend (2010)). If so, consider specifying this for the reader.

**Pg. 3**

- Consider including logarithmically spaced axis ticks for Fig. 1c. Both 1b and 1d have these ticks, and it makes their lack stand out for Fig. 1c.
- The authors used a linear heatmap to plot relative abundance in Fig. 1c. Since a number of macroecological patterns are examined using the logarithm of relative abundance, I wonder what this plot looks like with a logarithmic heatmap. Finally, the use of a diverging colormap is a strange choice. While coexistence requires strain B to have a relative abundance between zero and one, it is not clear why the authors chose to have the colormap diverge at 0.5. Did they run simulations for parameter regimes where their theory predicts that coexistence should occur at 0.5? If not, consider using a sequential colormap. Is so, it should be stated in the figure caption.

**Pg. 4**

“both strains could coexist, one can measure area of the space of parameters”

- Should it be “the area of the space”?

“Note that the width of this region depends only mildly (logarithmically) on the dilution factor D compared with its strong, inverse dependence on the resource concentration c0, suggesting that coexistence of strains should be possible for a broad range of environmental fluctuations”

- I would just say “logarithmically” here for *D* and replace “strong” with linear for c0.

**Pg. 5**

- Sub-figure label “c” is right in the middle of the figure.

**Pg. 7**

- Is there a reason why percentages are plotted on the barchart in Fig. 3e? Instead, I would recommend plotting 95% CIs of their simulation results.

**Pg. 8**

**“**investigating how nonlinearities in resource-dependent growth can lead to violations of the competitive exclusion principle”

- Specify where the nonlinearity comes from. My understanding is that they are referring to the form of the Monod growth equation, this should be made explicit.

**Pg. 9**

“we present every strain at equal amount of 1 unit at the beginning.”

- This description is unclear. Please explain what 1 unit is.

“Their maximum growth rates are 0.55 hr−1 and 1.0 hr−1 respectively, with substrate affinity being 0.01 a.u. and 4.0 a.u. In Fig.2b, we introduced an invader strain C whose maximum growth rates vary between [0.6, 0.7] hr−1 and substrate affinity vary between [0.25, 1.05] a.u.”

- Please briefly elaborate on the rationale behind these chosen values.

**Pg. 11**

**“**While in the case of the constant nutrient supply”

- Should it be “While in the case of constant nutrient supply”?

“can never exceed the number of (generalized) sources”

- Is “sources” supposed to be “resources”? And is “generalized” refer to “substitutable” in that two strains/species **can** consume the resource, though with different Monod parameters? If so, I would clarify that point.
- Please define c^{*} for the reader.

“under some conditions may make it D−fold simultaneously with B_{1}”

- Is this supposed to be “…may make it grow D−fold simultaneously….”?

“single source”

- Replace with “single resource”.

**Pg. 12**

- The caption for Fig. S1 needs additional detail.

**“**In (a), the variation in resource partitioning was as follows: each species’ resource partitioning was independently sampled from a normal distribution, N (0.2, 0.1 2 ) for species I and N (0.7, 0.1 2 ) for species II.”

- Is this the same variation in resource partitioning that was used for Fig. S2b? If so or if not, please provide this detail in the legend. Also, please briefly describe the change in the Monod parameters in the legend.

**Pg. 13**

“For the same reason the steady state initial abundance of the microbe for each dilution cycle should be…”

- This took me a few minutes to work out and I imagine it may also happen to a typical reader. Please provide additional detail on how you arrived at the solution for N_{10}.

**“**we can use (??) to give dc/dt as we …”

- Missing equation reference.

**Pg. 14.**

“…own growth rate by a tiny little bit….”

- What range of values is this?

**“**, whenever the nutrient supply come somewhere”

- Replace “come” with “is”.

**Pg. 15.**

“would have to concern oneself with the sign of the expression in the brackets, which we rewrite as”

- The expression is in parentheses, not brackets.

**Pg. 16.**

**“**That is what makes ∂f(x, y, D)/partialx from”

- “partialx” did not compile correctly.

**“**Rewrite (S11) in terms of x, y, γ. As, again..”

- It would be helpful for the reader if the authors described the motivation for this derivation.

**Pg. 17.**

- Please write out “l.h.s.” in Eq. S30.

“so that γ can be indeed coaxed into the interval between...“

- Do the authors mean to say that once can always choose some value of γ in this interval?

“that would be still a dynamics that is characterised by two parameters”

- I’d recommend replacing with “there would still be dynamics that is characterised by…”.

**Pg. 20.**

- For reference, it would be helpful for there to be a horizontal dashed grey line at 0.5 on the y-axis to remind the new reader about the meaning behind K_{A}^{Monod}.
- Fig. S4 is slightly cut-off on the y-axis.

Pg. 21

- “each providing its own value of the (exponential) growth rate for each microbe” It is unclear why “exponential” is in parentheses. Are the authors implying that their analysis is applicable to forms of growth rate functions beyond what they examined in this paper?
- “the number of survivors matching the number of sources” replace “matching” with “match”.
- “have different source preferences” should “source” be “resource”?
- “one of them starts consuming the resource number” remove “the”.
- “In our case, the species are bound to have the same food preferences, and in this case a multiple coexistence is much harder (though possible) to arrange.” Are the authors able to get some rough estimates on the degree of similarity of resource preferences? Is this basically a partition problem, where the
- Shorten “the species” to “species” and remove “a” from the sentence.

**References**

Baquero, F., Coque, T. M., Galán, J. C., & Martinez, J. L. (2021). The Origin of Niches and Species in the Bacterial World. *Frontiers in Microbiology*, *12*. https://www.frontiersin.org/article/10.3389/fmicb.2021.657986

Litchman, E., Edwards, K. F., & Klausmeier, C. A. (2015). Microbial resource utilization traits and trade-offs: Implications for community structure, functioning, and biogeochemical impacts at present and in the future. *Frontiers in Microbiology*, *6*, 254. https://doi.org/10.3389/fmicb.2015.00254

Muscarella, M. E., Howey, X. M., & Lennon, J. T. (2020). Trait-based approach to bacterial growth efficiency. *Environmental Microbiology*, *22*(8), 3494–3504. https://doi.org/10.1111/1462-2920.15120

Roller, B. R. K., Stoddard, S. F., & Schmidt, T. M. (2016). Exploiting rRNA operon copy number to investigate bacterial reproductive strategies. *Nature Microbiology*, *1*(11), 1–7. https://doi.org/10.1038/nmicrobiol.2016.160

Shoemaker, W. R. (2022). *A macroecological perspective on genetic diversity in the human gut microbiome* (p. 2022.04.07.487434). bioRxiv. https://doi.org/10.1101/2022.04.07.487434

Vellend, M. (2010). Conceptual Synthesis in Community Ecology. *The Quarterly Review of Biology*, *85*(2), 183–206. https://doi.org/10.1086/652373

Zhao, S., Lieberman, T. D., Poyet, M., Kauffman, K. M., Gibbons, S. M., Groussin, M., Xavier, R. J., & Alm, E. J. (2019). Adaptive Evolution within Gut Microbiomes of Healthy People. *Cell Host & Microbe*, *25*(5), 656-667.e8. https://doi.org/10.1016/j.chom.2019.03.007
